# Organ injury accelerates stem cell differentiation by modulating a fate-transducing lateral inhibition circuit

**DOI:** 10.1101/2024.12.29.630675

**Published:** 2024-12-30

**Authors:** Erin N. Sanders, Hsuan-Te Sun, Saman Tabatabaee, Charles F. Lang, Sebastian G. van Dijk, Yu-Han Su, Andrew Labott, Javeria Idris, Marco Marchetti, Shicong Xie, Lucy Erin O’Brien

**Affiliations:** 1 Department of Molecular and Cellular Physiology and Institute of Stem Cell Biology and Regenerative Medicine, Stanford University School of Medicine, Stanford, CA, USA.; 2 Department of Developmental Biology, Stanford University School of Medicine, Stanford, CA, USA.; 3 Department of Chemical and Systems Biology, Stanford University School of Medicine, Stanford, CA, USA.; 4 Eccles Institute of Human Genetics, University of Utah, Salt Lake City, UT, USA.; 5 Department of Biology, Stanford University, Stanford, CA, USA.; 6 Chan-Zuckerberg Biohub—San Francisco, San Francisco, CA, USA

## Abstract

Injured epithelial organs must rapidly replace damaged cells to restore barrier integrity and physiological function. In response, injury-born stem cell progeny differentiate faster compared to healthy-born counterparts, yet the mechanisms that pace differentiation are unclear. Using the adult Drosophila intestine, we find that injury speeds cell differentiation by altering the lateral inhibition circuit that transduces a fate-determining Notch signal. During healthy intestinal turnover, a balanced ratio of terminal (Notch-active) and stem (Notch-inactive) fates arises through canonical lateral inhibition feedback, in which mutual Notch-Delta signaling between two stem cell daughters evolves to activate Notch and extinguish Delta in exactly one cell. When we damage intestines by feeding flies toxin, mutual signaling persists, but a cytokine relay from damaged cells to differentiating daughters prevents the Notch co-repressor Groucho from extinguishing Delta. Despite Delta persistence, injured organs preserve the Notch-inactive stem cell pool; thus, fate balance does not hinge on an intact circuit. Mathematical modeling predicts that increased Delta prompts faster Notch signaling; indeed, in vivo live imaging reveals that the real-time speed of Notch signal transduction doubles in injured guts. These results show that in tissue homeostasis, lateral inhibition feedback between stem cell daughters throttles the speed of Notch-mediated fate determination by constraining Delta. Tissue-level damage signals relax this constraint to accelerate cell differentiation for expedited organ repair.

## Introduction

Mature organs must respond to unpredictable environmental insults throughout an animal’s lifetime. For barrier epithelial organs, the need to quickly regenerate damaged cells following such insults is acute because damage to the barrier compromises the integrity of the body. In response to injury, adult epithelial stem cells accelerate the rate of replacement divisions. These new stem cell progeny cannot form an effective barrier, however, because they are born in an undifferentiated state. This predicament raises the question of whether injury-born cells differentiate at an accelerated pace.

Longstanding observations suggest that injury can indeed drive stem cell progeny to differentiate faster. In the barrier epithelia that line the mammalian and adult *Drosophila* intestinal tract^1–6^ as well as mammalian airway^7,8^and skin^9,10^, stem cell progeny in damaged tissues acquire morphological and transcriptional maturity in less time compared to undamaged tissues. Expediting the differentiation of new stem cell progeny should restore the barrier and other physiological functions to damaged tissues more rapidly. Moreover, it would prevent the accumulation of excess undifferentiated cells that otherwise might predispose to disease.

Cell differentiation is instructed by fate signals, and the identities of these signals are unchanged by injury. In principle, faster transduction of fate signals might provide the impetus for faster cell differentiation. Direct evidence for this model is wanting, however, and, although much is known about how injury alters the milieu of signaling factors available to cells, how injury might modulate the speed at which signal transduction occurs is unclear.

**Figure 1.**
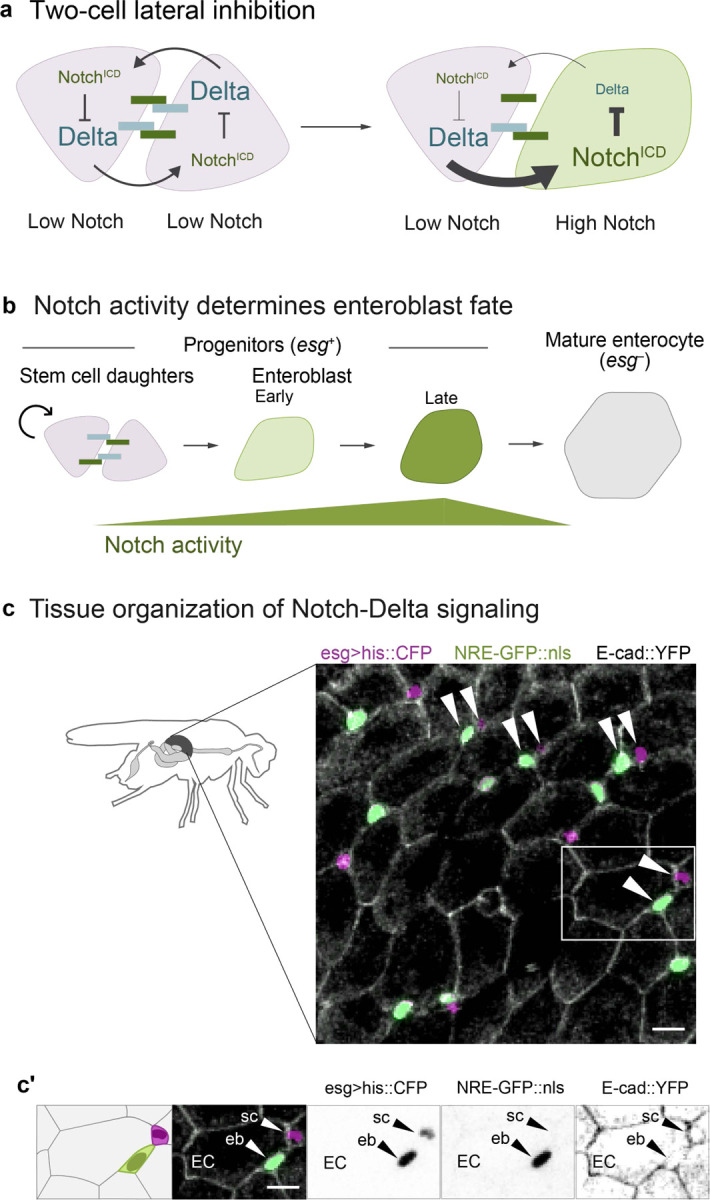
Notch-Delta signaling in the *Drosophila* adult midgut. (a) Two-cell lateral inhibition through Notch-Delta signaling. Initially, both cells express Notch receptor (dark green) and Delta ligand (blue). Stochastic differences in the two cells’ signaling levels are amplified through a feedback circuit in which Notch-Delta trans-activation and release of the Notch intracellular domain (Notch^ICD^) results in downregulation of Delta (Extended Data Fig 1a). Over time, this circuit resolves into opposing cell states of high Notch, low Delta and low Notch, high Delta. (b) Notch-Delta fate specification in the absorptive lineage. New mitotic stem cell daughters (pink) engage in mutual Notch-Delta signaling. Cell fate is determined by Notch activity: daughters that remain at sub-threshold Notch activity remain stem cells, while those that exceed the threshold differentiate into enteroblasts (early: light green; late: dark green). Enteroblasts progressively mature into terminal enterocytes (gray). The immature progenitor population (stem cells and enteroblasts) is marked by *escargot* (*esg*). (c) Tissue organization of Notch-Delta signaling. Small progenitor cells (*esg>his2b::CFP*, magenta) are interspersed among large enterocytes (outlined by *ubi-E-cad::YFP*, grayscale). Notch activity is visualized using the *NRE-GFP::nls* reporter (green; Fig S1b). Progenitors frequently form pairs of one GFP^+^ and one GFP^−^ cell (arrowheads). Both GFP^+^ and GFP^−^ cells are *esg*^+^, although GFP^+^ cells appear light green in the overlay. Scale bar, 10μm. (c’) Single-channel views of a representative cell pair (white box in c) demonstrate *esg* expression in GFP^+^ and GFP^−^ cells. Scale bars, 10μm.

We examined these issues following injury of the intestinal epithelium that lines the adult *Drosophila* midgut. In the fly gut, as in many mammalian organs including skin, airway, and mammary gland, cell differentiation is instigated by Notch receptor activation^7,11–19^. During tissue homeostasis, signaling occurs via a lateral inhibition circuit between stem cell daughter pairs^20–22^: Delta ligand on one cell activates Notch on its partner, which causes the partner cell to downregulate Delta (Fig. 1a) (ref- lateral inhibition reviews). Over time, this circuit resolves to generate a balanced ratio of terminal (Notch-active) and stem (Notch inactive) fates; hence, it was assumed that ensuring proper fate balance was its primary function^20–22^. Intriguingly, however, we find that in injured guts—which maintain balanced division fate outcomes^23^—Notch activation no longer yields Delta downregulation. Thus, lateral inhibition is dispensable for fate balance.

Instead, we find that this injury-altered circuit drives faster signaling. Uncoupling Delta downregulation from Notch activation results in a higher level of Delta, which in turn accelerates real-time Notch signal activation. This rewiring is a consequence of phospho-inactivation of Groucho, a Notch co-repressor that controls Delta transcription^22,24–26^, and is triggered by cytokines released from damaged intestinal enterocytes.

Thus, in tissue homeostasis, lateral inhibition feedback throttles the speed of Notch-regulated cell differentiation by limiting Delta ligand. Tissue injury opens this throttle by deploying damage signals that remove this kinetic limiter. The consequently accelerated tempo of differentiation works in concert with faster stem cell divisions to expedite production of mature, physiologically functional cells that the injured tissue needs.

## Background

In the fly intestinal epithelium, the gut’s enterocyte lineage, which accounts for >90% of midgut cells, comprises just three, ontogenically linked cell types: stem cells, enteroblasts, and enterocytes (Fig. 1b, 1c). Stem cells both self-renew and generate enteroblasts, which are post-mitotic precursors that mature directly into enterocytes. These terminal enterocytes are polarized epithelial cells that form the intestinal barrier and secrete digestive enzymes. Unlike the mammalian intestine’s crypt-villus architecture, fly stem cells and enteroblasts are dispersed among the much-larger enterocytes (Fig. 1c). Midgut stem cells and enteroblasts are collectively termed progenitors and are marked by the transcription factor Escargot (Esg) (Fig. 1b).

The stem-to-enteroblast transition offers a uniquely tractable system to study Notch fate regulation in maturity. While most *in vivo* models involve multicellular fields with multiple receptors and ligands, the stem-to-enteroblast transition typically occurs in isolated, two-cell pairs and involves a single receptor-ligand complex (Fig. 1c). Both stem cells and newborn daughters express the Notch receptor and its ligand Delta. When these cells contact—either post-division or through physical collision^27^—they engage in juxtacrine signaling (Fig. S1a). This sets into motion a feedback loop that resembles a classic lateral inhibition circuit (Fig. 1a)^20–22,28^: Cells that accumulate sufficient Notch activity become enteroblasts and, by downregulating Delta, maintain their partners as Notch-inactive stem cells. Eventually, the enteroblasts will themselves attenuate Notch as they mature into large, terminal enterocytes (Fig 1b)^13^.

**Fig. 2.**
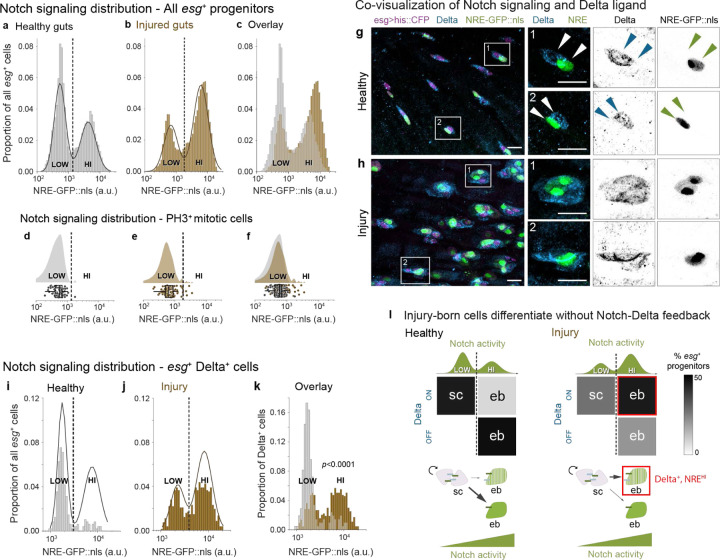
Injury disrupts Notch-Delta lateral inhibition feedback while maintaining cell fates. (a-c) Notch signaling (NRE-GFP::nls) in progenitors (*esg>his2b::CFP*) from (a) healthy and (b) bleomycin-injured guts. Both conditions show bimodal NRE^low^ and NRE^hi^ populations (solid lines: Gaussian mixture model (GMM) fits; dashed lines: classification thresholds). (c) Overlay shows injury increases the proportion of NRE^hi^ cells while maintaining GFP intensity ranges and thresholds. Healthy: n=5681 cells, N=6 guts. Injury: n=8819 cells, N=6 guts. (d-f) NRE-GFP::nls in mitotic (PH3^+^) cells shown as raincloud plots (top) and single-cell measurements (bottom) from (d) healthy and (e) injured guts. Dashed lines show classification thresholds from panels a-b. In both conditions, PH3^+^ cells match the NRE^low^ peak distribution and classification (healthy: 98% NRE^low^; injured, 93% NRE^low^). (f) Overlay. Healthy: n=60 cells, N=27 guts. Injury: n=83 cells, N=8 guts. (g-h) Co-visualization of Notch signaling (*NRE-GFP::nls*, green) and Delta immunostain (blue) in *esg>his2b::CFP* progenitors (magenta). (g) In healthy guts, Delta^+^ cells typically lack GFP and pair with Delta^–^, GFP^+^ cells. (h) In injured guts, many Delta^+^ cells show bright GFP and often form clusters with other Delta^+^, GFP^+^ as well as Delta^+^, GFP^–^ cells. Boxed regions shown at higher magnification with split channels. Scale bars, 10μm. (i-k) Quantification of Delta and Notch signaling relationships. Notch signaling (NRE-GFP::nls) specifically in Delta^+^ cells from (i) healthy and (j) injured guts, as a proportion of all *esg*^+^ cells. Solid lines: GMM fits for all *esg*^+^ population. NRE-GFP::nls raw values and classification thresholds (dashed lines) differ from panels a-c due to use of a different imaging system (see Methods). Overlay of Delta^+^ cells from (i) healthy and (j) injured as a proportion of Delta^+^ cells only. Injury shifts Delta^+^ cells from predominantly NRE^low^ (84%) to predominantly NRE^hi^ (62%) (p<0.0001). Healthy: n=478 *esg*^+^ cells, n=208 Delta^+^ cells; N=2 guts. Injured: n=823 *esg*^+^ cells, n=631 Delta^+^ cells; N=3 guts. p-value, two-sample K-S test. (l) Summary: Injury-born cells differentiate in the absence of Notch-Delta feedback. In healthy guts, mitotic stem cells (sc) express Delta and maintain low Notch activity, while lateral inhibition feedback drives differentiating enteroblasts (ebs) to the opposing state of high Notch activity and no Delta. In injury, differentiating enteroblasts maintain Delta despite acquiring high Notch. Gray shading indicates percent of progenitors in each Notch/Delta state; green curves show GMM NRE-GFP::nls distributions (Fig 2a–c). Progenitors lacking both Delta and GFP were excluded from quantitation.

To quantify Notch activity, we measured single-cell intensities of the sensitive reporter NRE-GFP::nls (Fig S1b)^20,27^. Using *esgGAL4;UAShis2b::CFP* (hereafter, *esg*) to identify progenitors in healthy guts^29^, we confirmed that the GFP intensities of *esg*^+^ cells form a sharp bimodal distribution (Fig 2a), as previously reported^27^. Retrospective analysis of long-term live movies showed that these two populations correspond to stem cells and enteroblasts respectively^27^. As an incipient enteroblast activates Notch, its *NRE-GFP::nls* intensity ‘moves’ over time from NRE^low^ to NRE^hi^; the stem-to-enteroblast transition takes place when the cell’s Notch activity level crosses the trough separating the two states^27^, which we designate NRE^low^ and NRE^hi^.

## The Notch threshold for terminal fate specification remains constant in injury

One potential mechanism to accelerate differentiation during injury would be to make the enteroblast differentiation program more sensitive to Notch signaling, such that injury-born cells acquire enteroblast fate at a lower level of Notch activity compared to healthy-born cells. Heightened sensitivity to Notch would manifest, for example, as a leftward shift in the position of the trough between NRE^low^ (stem) and NRE^hi^ (enteroblast) peaks.

To investigate this possibility, we compared the population-scale distribution of Notch signaling in healthy and injured states. We induced injury by feeding flies bleomycin during days 3–4 of adult life. Bleomycin is a DNA-damaging agent that targets mature enterocytes while sparing progenitor cells^4^. At the moderate concentration (25 μg/ml) we used, barrier integrity and organismal survival are not impacted during the two-day duration of these injury experiments^4^. Bleomycin treatment dramatically increased the number of *esg*^+^ progenitor cells per gut, as expected from damage-induced regeneration^4,30^.

As with healthy guts (Fig. 2a), we measured NRE-GFP:nls intensities in individual *esg*^+^ cells in injured guts (Fig. 2b). The GFP distribution remained bimodal, with distinct NRE^low^ and NRE^hi^ populations. We observed that injury increased the proportion of NRE^hi^ cells (Fig 2b), consistent with rapid production of replacement cells during regeneration (refs). Yet despite this proportional shift, Gaussian Mixture Model (GMM) analysis reveals that injury preserves fundamental features of the NRE^low^ and NRE^hi^ states: The overall range of GFP intensities, the modes of both populations, and the position of the trough (decision boundary) between the populations all remain similar to healthy guts (Fig. 2c). Thus, while injury shifts the distribution of cells across the two Notch signaling states, it does not fundamentally alter the states themselves.

We next compared the Notch activity level at which cells transition from stem cells to enteroblasts by using mitotic activity as an orthogonal identifier of stem cells. Mitoses are virtually exclusive to stem cells in both healthy^12,13,29,31–33^ and bleomycin-injured guts^32^. We identified mitotic cells by immunostaining for the M-phase marker phospho-Histone H3 (PH3). As expected from prior reports of damage-induced stem cell hyperproliferation^4,6,30,32,34–41^, injured guts contained markedly greater numbers of PH3^+^ cells. We measured the *NRE-GFP:nls* intensities of individual PH3^+^ cells in healthy and injured guts and compared these to the corresponding all-progenitor GFP distributions (Fig 2d, 2e).

These comparisons revealed that the stem-to-enteroblast transition occurs at a near-identical *NRE-GFP:nls* intensity in healthy and injured guts. First analyzing healthy guts, we found that nearly all (98%) PH3^+^ cells were NRE^low^ (Fig. 2d). Further-more, the shape of the healthy-gut PH3^+^ cell distribution (Fig. 2d) virtually matches that of NRE^low^ cells in the all-progenitor distribution (Fig. 2a). These patterns corroborate prior live imaging (Martin 2018) and confirm that the trough between NRE^low^ and NRE^hi^ represents the Notch signaling level at which cells become enteroblasts.

Next analyzing injured guts, we found this pattern was upheld: 93% of PH3^+^ cells were NRE^low^ (Fig. 2e), and the GFP distribution of injured-gut PH3^+^ cells (Fig. 2f) again resembles that of the all-progenitor NRE^low^ population (Fig. 2b). These data demonstrate that mitotic behavior remains tightly associated with the NRE^low^ state in injury. Since the threshold GFP intensity that separates NRE^low^ and NRE^hi^ is the same in injured and healthy guts (Fig. 2c), then by implication, cells become enteroblasts at the same Notch signaling level. We conclude that injury does not alter the level of Notch signaling required for enteroblast fate and that injured guts must use other mechanisms to accelerate enteroblast differentiation.

## Notch-Delta feedback is disrupted in injury

An alternative scenario is that accelerated differentiation arises from injury-induced changes to Delta ligand. To explore this notion, we first characterized the relationship between Delta expression and Notch activation during tissue homeostasis. Three lines of evidence demonstrated that individual cells either express Delta or activate Notch signaling—but not both: First, immunostaining for Delta protein in healthy guts of genotype *NRE-GFP::nls, esg>his2b::CFP* showed that Delta^+^ cells typically lacked GFP and were frequently paired with Delta^−^ cells that exhibited bright GFP (Fig. 2g), as has been reported previously^20–22^. Second, GFP measurements demonstrated that the vast majority (84%) of Delta^+^ cells were NRE^low^ (Figs. 2i, 2j) and that a similar majority (86%) of NRE^hi^ cells were Delta^−^ (Fig. S2a, S2b). Third, our analysis of published single-cell transcriptomes^42^ revealed strong anti-correlation between Delta ligand and Notch target gene expression (Fig. S1c–f). Altogether, these data exemplify two-cell lateral inhibition: Cells express Delta until they reach the precise threshold of Notch activity marked by the trough between NRE^low^ and NRE^hi^ peaks; at this point, they simultaneously turn off Delta and become enteroblasts.

Injury dramatically altered the relationship between Delta expression and Notch activation. In sharp contrast to healthy guts, progenitors in injured guts showed widespread co-expression of Delta and NRE-GFP:nls (Fig. S2b): In injured *NRE-GFP::nls, esg>his2b::CFP* guts, immunostaining revealed numerous Delta^+^ cells with bright GFP signal^43^ (Fig. 2h). These cells often formed clusters with other Delta^+^, GFP-expressing cells and with Delta^+^ cells that lacked GFP (Fig. 2h)^43^. Measuring single-cell GFP intensities, we found that 62% of Delta^+^ cells were NRE^hi^ (Fig 2k, 2l)—a striking, four-fold increase compared to healthy guts. Correspondingly, proportions of Delta^+^, NRE^low^ cells and Delta^−^, NRE^hi^ cells dropped by 60% and 49%, respectively (Fig. 2m, 2n, and S2b). The dramatic emergence of dual, Delta^+^, NRE^hi^ cells indicates that injury uncouples Delta downregulation from Notch activation, disrupting the feedback circuit that normally drives cells toward opposing signaling states (Fig. 2o).

Our Fig 2a–e results reveal the identity of this dual Delta^+^, NRE^hi^ population. Since NRE-GFP:nls levels reliably distinguish cell fates even during injury—with NRE^hi^ marking enteroblasts and NRE^low^ marking stem cells—we conclude that these Delta^+^, NRE^hi^ cells are enteroblasts that fail to downregulate Delta (Fig. 2o). This persistent expression of Delta in most injury-born enteroblasts demonstrates widespread loss of Notch-Delta feedback in injured guts. (Incidentally, these data also imply that Delta immunostaining, which is conventionally used to mark stem cells in healthy guts, no longer distinguishes stem cells from enteroblasts after injury.) Yet despite pervasive loss of feedback, 38% of Delta^+^ cells remain NRE^low^ (Fig. 2l) and thus maintain stemness, a finding consistent with twin-spot MARCM evidence that asymmetric division fates remain prevalent in injury^23^. It is currently unclear how injured-gut stem cells selectively escape Notch activation. Nonetheless, robust maintenance of an NRE^low^ population is crucial to avoid exhaustion of the stem cell pool. Most importantly, these findings imply that Notch-Delta lateral inhibition feedback—traditionally considered the basis for asymmetric fate determination—is dispensable for specifying binary fates.

## Modeling links Notch-Delta feedback to Notch signaling speed

**Figure 3:**
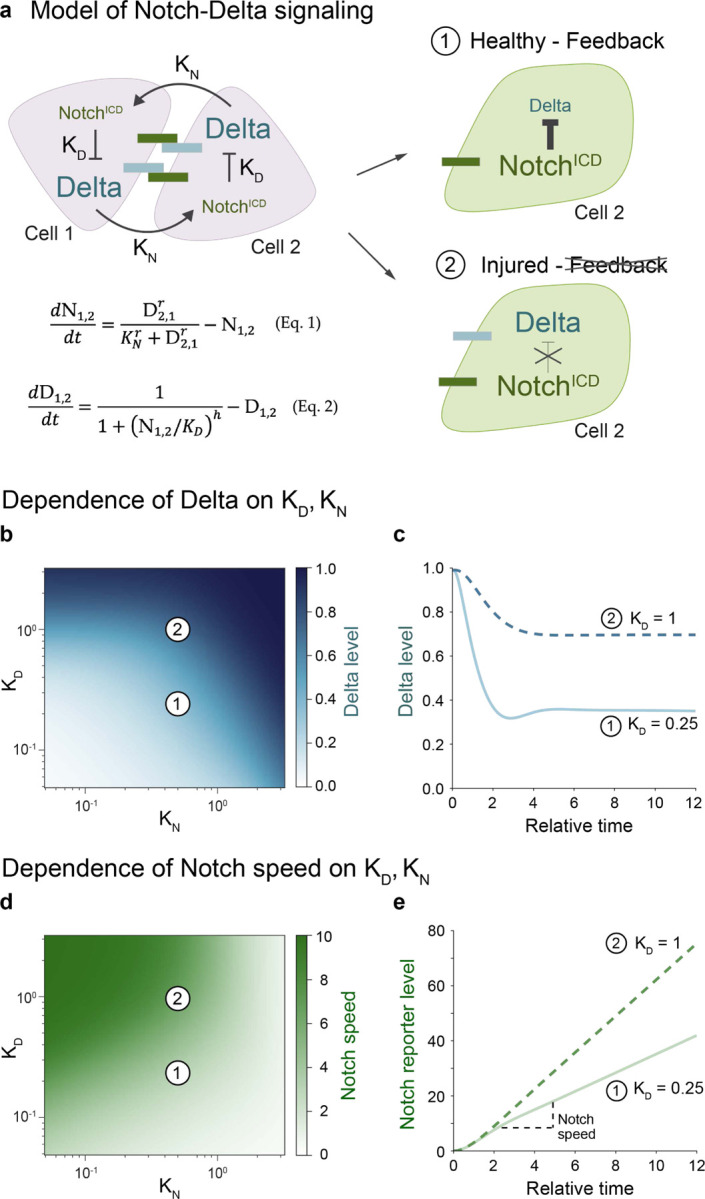
Disrupted Notch-Delta feedback can accelerate Notch signaling. (a) Model schematic for Notch-Delta lateral inhibition (Collier 1996, Guisoni 2017). Two key parameters govern the system: K_N_ (the threshold for Notch activation by Delta) and K_D_ (the threshold for Delta inhibition by Notch). Cell 2 is initialized with slightly higher Notch activity. Outcomes 1 (high-Notch/low-Delta) and 2 (high-Notch/high-Delta) represent the dominant enteroblast states in healthy and injured guts, respectively. Equations 1–2 describe the time evolution of Notch activity and Delta levels. Hill coefficients r=h=2. (b-d) Model parameter space and dynamics. Parameter values for Point 1 (K_N_=0.5, K_D_=0.25); Point 2 (K_N_=0.5, K_D_=1). (b) Steady-state Delta level (t=12) as a function of K_N_ and K_D_. While injury decreases K_N_ and increases K_D_ (see Results), only increased K_D_ reproduces the high-Notch/high-Delta injury state. (c) Simulated time evolution of Delta levels for Points 1 and 2. See Fig. S4a for additional K_D_ values. (d) Notch signaling speed as a function of K_N_ and K_D_. Signaling speed is defined as the mean rate of Notch reporter accumulation from t=2 to t=12. Increased K_D_ accelerates signaling speed. (e) Simulated time evolution of Notch reporter levels for Points 1 and 2. See Fig. S4b for additional K_D_ values.

We wondered whether disrupted Notch-Delta feedback underlies faster Notch-driven fate signaling in injury. To examine this possibility, we used a mathematical model of lateral inhibition in which transactivation of Notch by its partner’s Delta is coupled to same-cell inhibition of Delta by activated Notch^ICD 28^ (Fig. 3a; see Methods). The model is governed by two dimensionless parameters: K_N_, which is the threshold for Notch activation by Delta, and K_D_, which is the threshold for Delta inhibition by Notch^ICD^ (Fig. 3a). Both cells initially have high Delta and low Notch, with symmetry broken by a slight elevation of Notch in one cell. The time evolution of Notch activity and Delta level is defined by Eqs. 1 and 2 (Fig. 3a) using experimentally-derived parameter ranges from healthy guts^21^ (see Methods).

We first sought to identify model parameters that reproduce the injury-induced high-Notch/high-Delta state. Since K_N_ is inversely proportional to cell-cell contact area^21^, and contact area increases in injury^43^ (compare Fig. 2g,h), we predicted that injury would decrease K_N_. However, reducing K_N_ in our simulations failed to produce an injury-like state—instead of maintaining high Delta, cells with high Notch showed reduced Delta (Fig. 3b). This result persisted in a three-cell model simulating injury-induced clusters (see Modeling Supplement). Thus, changes in K_N_ alone cannot explain the injury phenotype.

We then examined K_D_, which is inversely related to Notch^ICD^’s ability to suppress Delta. Since many high-Notch cells continue to express Delta during injury, K_D_ is presumably increased. Indeed, increasing K_D_ in both two- and three-cell simulations resulted in high-Notch cells with elevated Delta, reproducing the injury state (Fig. 3b, 3c; Modeling Supplement).

**Figure 4:**
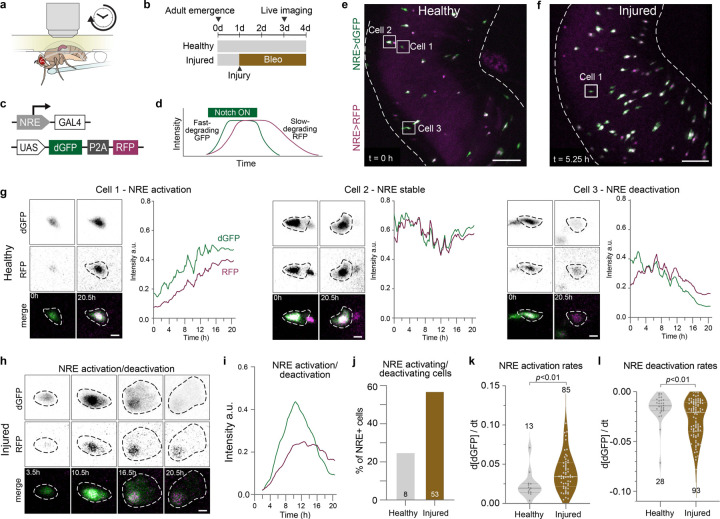
Injury accelerates Notch signal activation and deactivation (a) Schematic of long-term live imaging setup (Martin 2018). The R4ab region of the midgut is imaged overnight through a “window” cut in the cuticle of the living adult fly. Flies feed through a microcapillary tube. (b) Feeding scheme for live imaging experiments. Adult females are collected on day 0 post-eclosion and placed in vials with males. For the injury condition, flies are fed 25ug/mL bleomycin in yeast paste for 48 hours prior to imaging. Guts are imaged starting adult day 3. (c) Schematic of TransTimer multi-cistronic genetic construct. The Notch response element GBE-Su(H)-GAL4 (NRE) drives GAL4-mediated expression of a fast-folding, fast-degrading GFP (dGFP) as well as a slower-folding, slow-degrading RFP, connected by a P2A peptide. (d) Schematic of hypothetical NRE>TransTimer dGFP and RFP intensity traces in response toactivation and deactivation of Notch. (e) Representative still of a healthy live-imaged NRE>TransTimer gut. Boxed cells correspond to representative cells in (g). Scale bar: 50μm. See also Movie 1. (f) Representative still of an injured live-imaged NRE>TransTimer gut. Boxed cell corresponds to representative cell in (h). Scale bar: 50μm. See also Movie 2. (g) Representative stills of three healthy progenitor cells from (e) and their TransTimerGFP/RFP traces through 1-NRE activation: increasing TransTimerGFP and RFP intensity; 2-NRE stable: flat TransTimer traces; and 3-NRE deactivation: decreasing TransTimer intensity. Scale bars: 5μm. See also Movies 3–5. (h) Representative stills of an injured progenitor cell from (f) going through both NRE activationand deactivation stages in the course of a single movie. Scale bar: 5μm. See also Movie 6. (i) Representative TransTimerGFP/RFP traces for an injured progenitor cell from (f) goingthrough NRE activation and deactivation in the course of a single movie. (j) Quantification of percentage of NRE+ cells that go through both NRE activation anddeactivation stages in the course of a single movie for both healthy and injured conditions. Healthy: 25%; n=8 cells; N=2 guts. Injured: 57%; n=53 cells; N=3 guts. (k) Quantification of rates of Notch activation by measuring the slopes of the increasing portionsof NRE>TransTimerGFP cell tracks. Notch activation is roughly twice as fast in injured guts compared to healthy controls. Healthy: n=13 cells; N=2 guts. Injured: n=85 cells; N=3 guts. Horizontal lines represent median and 25th, 75th percentiles. *p-*values and medians, Mann-Whitney test. (l) Quantification of rates of Notch deactivation by measuring the slopes of the decreasingportions of NRE>TransTimerGFP cell tracks. Notch deactivation is nearly twice as fast in injured guts compared to healthy controls. Healthy: n=28 cells; N=2 guts. Injured: n=93 cells; N=3 guts. Horizontal lines represent median and 25th, 75th percentiles. *p-*values and medians, Mann-Whitney test.

Having identified increased K_D_ as a key parameter change, we next investigated its effect on Notch signaling dynamics. When K_D_ is elevated, cells maintain higher Delta levels during Notch-Delta signaling, potentially providing more ligand to activate Notch. We hypothesized this would accelerate Notch target gene accumulation and thus cell differentiation. To test this, we added a Notch^ICD^-driven reporter to our model (see Methods) and calculated Notch signaling speed as the rate of reporter accumulation. Consistent with our hypothesis, increased K_D_ led to faster Notch signaling during the initial, linear phase of signaling across a broad range of K_NS_ (Fig. 4d, e). Overall, these analyses predict that disrupted Notch-Delta feedback accelerates Notch signaling speed.

## Notch signal activation and deactivation both accelerate in response to injury

We examined this prediction by performing real-time imaging of single-cell Notch dynamics in healthy and injured guts *in vivo*. We opened a viewing window in the animal’s dorsal cuticle (Fig 4a), enabling imaging of the midgut in awake, moving flies^27^. Using this ‘Windowmount’ protocol, flies continue to ingest food and defecate throughout imaging, and the GI tract, with all its associated tissues including neurons, trachea, immune cells, and fat, remain physiologically functional for up to 20 hours^27^.

To monitor Notch signaling in single differentiating cells with high temporal resolution, we expressed a dual-color kinetic reporter (UAS-TransTimer)^44^ under control of the Notch Response Element (NRE-GAL4)^45^ (Fig. 4b). The TransTimer’s fast-folding, destabilized dGFP (maturation ~0.1 h; half life ~2h)^44^ sensitively reports changes in NRE-GAL4 activity. By contrast, its slow-folding, long-lived RFP (maturation ~1.5 h; half life ~20 h)^44^ persists in cells after Notch deactivation; these cells, which are in later stages of the enteroblast-to-enterocyte transition (Fig. 1b), exhibit RFP but not GFP signal.

We acquired two-channel Windowmount movies of NRE-driven TransTimer (hereafter NRE>TransTimer) in both healthy and injured midguts of 3-day old adults (Fig. 4d, 4e; Movies 1–2). Our imaging strategy generated high-quality, single-cell data by combining three key features: (1) organ-scale, volumetric imaging (~250×250×150 μm) for unbiased, simultaneous capture of multiple NRE>TransTimer cells per gut; (2) micron-level spatial resolution for precise 3D segmentation; and (3) frequent time points (every 7.5 minutes) over 20-hour sessions for high temporal resolution during biologically meaningful timespans. We traced individual NRE>TransTimer cells from their first appearance until either signal loss or the end of imaging. At each timepoint, single-cell GFP and RFP intensities were quantified (see Methods).

Analysis of the resulting single-cell traces revealed four NRE activity patterns: activation, stability, deactivation, and activation→deactivation (Fig 4f–i; Movies 3–6). Strikingly, in injured guts, most tracked cells (55%) underwent activation→deactivation transitions—exceeding the other three categories combined (Fig. 4j). In healthy guts, by contrast, only 25% of cells exhibited activation→deactivation transitions. Activation→deactivation traces displayed the expected temporal offset between dGFP and RFP dynamics; on the other hand, other traces typically showed little or no offset, likely because NRE dynamics changed on a timescale similar to or slower than the ~20-hour half-life of RFP. These real-time TransTimer traces provide ground-truth data for the interpretation of TransTimer fluorescence in fixed analyses dependent on endpoint GFP:RFP ratios. Overall, the prevalence of activation→deactivation transitions in injured, but not healthy guts, implies that injury accelerates Notch signaling.

Next, we took advantage of the sensitive measurements of fast-folding TransTimerGFP fluorescence to precisely calculate real-time Notch signaling speed by measuring the slope of NRE>TransTimerGFP tracks (see Methods). In definitive support of the prediction from modeling that injury-mediated disruption of lateral inhibition results in faster Notch signaling, we found that the rate of increase of NRE>TransTimerGFP is almost two-fold higher in injured guts than control (Fig 4p). Similarly, the rate of NRE>TransTimerGFP decrease is nearly twice as fast in injured guts than controls (Fig 4q). Thus, injured progenitors are not only traveling through Notch activation and deactivation stages more frequently, but their rates of Notch activation and deactivation are considerably accelerated.

These data show the first real-time, single-cell kinetics of a fate-specifying signal in a live adult organ. We now have the unprecedented view that stem cell daughters are not only generated faster following tissue damage, but that the speed of the Notch signals governing their fate outcomes is explicitly accelerated. This, in conjunction with our characterization of the modulation of lateral inhibition in injured tissues, describes a mechanism by which fate-determining signaling circuits can be flexibly adjusted to ramp up new mature cell generation and support rapid organ repair.

## Injury-induced inactivation of the Groucho co-repressor underlies loss of Notch-Delta feedback

The notion that higher K_D_ underlies injury-induced disruption of lateral inhibition aligns with our in vivo findings that the activity of the E(spl) co-repressor Groucho (Gro) is both essential to turn off Delta in NRE^hi^ cells during homeostasis and sufficient to re-establish Delta downregulation in NRE^hi^ cells during injury (Fig. 2). We propose that injury-induced disruption of lateral inhibition occurs by raising K_D_ through disruption of Gro-mediated Delta repression.

Groucho is a global transcriptional corepressor which acts to regulate Notch signal transduction in conjunction with the Hairless-Su(H) complex^46^ (Fig S1a). Following Notch activation, Gro interacts and cooperates with Notch transcriptional targets such as the E(spl)-C proteins^26^. In the *Drosophila* midgut, Gro functions as a corepressor for E(spl)-C to suppress Delta expression, inhibit cell-cycle re-entry, and facilitate cell differentiation in enteroblasts^22^.

**Figure 5.**
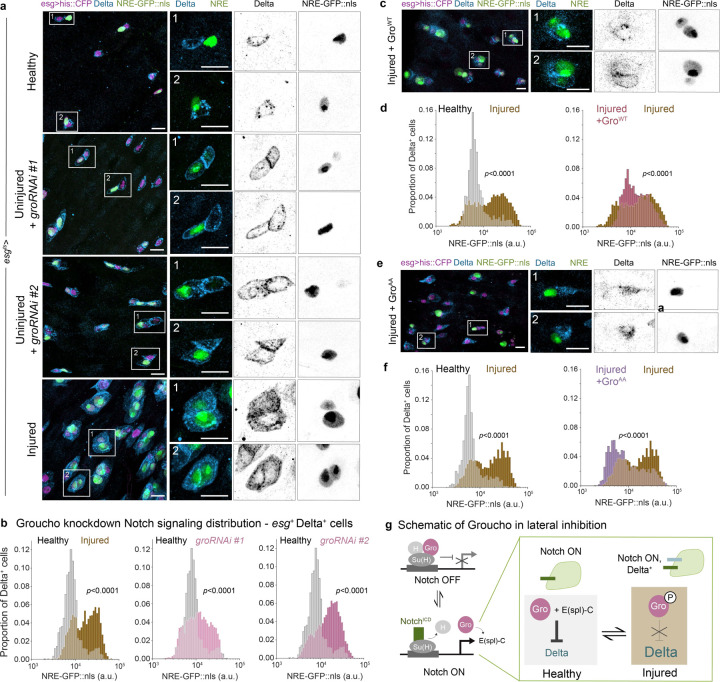
Groucho is necessary and sufficient to maintain Notch-Delta lateral inhibition (a) Progenitor cells (*esg>his2b::CFP*, magenta) in healthy (4-day) guts, uninjured guts with *esg*^*ts*^ driving *groucho* RNAi for adult days 0–4, and injured guts (bleomycin ingestion for adult days 3–4). Nearly all progenitors in both lines of *groRNAi* knockdown guts express Delta (anti-Delta immunostain, blue), with or without NRE-GFP::nls expression (green). *groRNAi* #1: VDRC #KK110546. *groRNAi* #2: BDSC #91407. Scale bars: 10 μm. (b) Comparison of single-cell Notch signaling distributions for all Delta+ cells in healthy, uninjured + *groRNAi*, and injured guts. Histograms show single-cell NRE-GFP::nls intensities for all Delta^+^, *esg>his2b::CFP* cells in the gut R4ab region. Large proportions of Delta^+^ cells shift to the NRE^hi^ peak when *gro* is depleted. Delta^+^ cells identified by immunostaining. Healthy: n=1328 cells; N=7 guts. Uninjured + *groRNAi* #1: n=4766 cells; N=14 guts. Uninjured + *groRNAi* #2: n=6945 cells; N=14 guts. Injured: n=2251 cells; N=6 guts. *p*-values, two-sample K-S test. See also Figures S2c, S5b. (c) Progenitor cells in injured guts with *esg*^*ts*^*>UAS-gro*^*WT*^ (overexpression of wildtype groucho). Prevalence of large multi-cell Delta^+^ progenitors is reduced, as is overall Delta expression (anti-Delta immunostain), though many individual cells still exhibit both *NRE-GFP::nls* and Delta. Scale bars: 10 μm. (d) Comparison of Notch signaling distributions for all Delta^+^ cells in healthy, injured + *esg*^*ts*^*>UAS-gro*^*WT*^, and injured guts. Some proportion of Delta^+^ NRE^low^ cells in injured guts is restored by gro^WT^ overexpression. Healthy: n=821 cells; N=7 guts. Injured + *gro*^*WT*^: n=738 cells; N = 11 guts. Injured: n=2814 cells; N=5 guts. *p*-values, two-sample K-S test. See also Figures S2d, S5c. (e) Progenitor cells in injured guts with *esg*^*ts*^*>UAS-gro*^*AA*^ (overexpression of phosphorylation-resistant groucho). Progenitors rarely form multi-cell clusters, and fewer individual cells exhibit both *NRE-GFP::nls* and Delta. Scale bars: 10 μm. (f) Comparison of Notch signaling distributions for all Delta^+^ cells in healthy, injured + esg^ts^>UAS-*gro*^*AA*^, and injured guts. The proportion of Delta^+^ NRE^low^ cells in injured guts is largely restored by gro^AA^ overexpression. Healthy: n=1083 cells; N=5 guts. Injured + *gro*^*AA*^: n=2119 cells; N=11 guts. Injured: n=2581 cells; N=5 guts. *p*-values, two-sample K-S test. See also Figures S2e, S5d. (g) Schematic of how Groucho’s function is modulated in injured vs healthy guts. In the absence of Notch^ICD^, Gro complexes with Hairless (H) and Suppressor of Hairless (Su(H)) as a corepressor of Notch target genes. When Notch^ICD^ is activated and binds to Su(H), Notch targets such as the Enhancer of split complex (E(spl)-C) are transcribed. In healthy guts, Gro then works with E(spl)-C to repress Delta in the now Notch-ON cell. However, in injury, Gro can be phosphorylated to downregulate its function, thus releasing repression of Delta and attenuating lateral inhibition feedback leading to Delta^+^ NRE^hi^ cells.

We were struck by prior work that showed depleting *gro* in *Drosophila* gut progenitor cells led to the accumulation of Delta^+^ cells and disrupted lateral inhibition^22^, reminiscent of the Delta^+^ NRE^hi^ cells we see in injury. We performed a similar experiment with two independent *groRNAi* lines driven by the progenitor-specific driver esgGAL4 with the temperature-sensitive repressor GAL80^ts^ in the background (esgGAL4; tubGAL80^ts^ – hereafter, *esg*^*ts*^). In uninjured guts with Gro knockdown, virtually all esg^+^ progenitors (visualized by UAS-his2b::CFP) stain strongly for Delta (Fig 5a), regardless of their Notch activity (as identified by NRE-GFP:nls expression). Indeed, we quantify over 86% of all esg^+^ cells are Delta^+^ in both Gro knockdown conditions (Fig S2c). Amongst the Delta-expressing populations, a large proportion (31% and 52%, respectively) correspond to NRE^hi^ cells (Fig S2c). Conversely, quantifying the proportion of NRE^hi^ cells that are Delta^+^ in Gro-depleted guts reveals that ~85% of enteroblasts now retain Delta expression (Extended Data Figs S2c, S5b). Therefore, the increase in Delta^+^ cells in Gro-depleted guts can be predominantly attributed to NRE^hi^ enteroblasts, confirming a requisite role for Gro in coupling Notch activation to Delta downregulation.

## Ectopic Groucho re-establishes injury-disrupted Notch-Delta feedback

Given that Gro is necessary for coupling Notch activation to Delta repression under homeostasis, we asked whether and how its activity may be altered during injury to modulate lateral inhibition circuitry. Importantly, it has been reported in other tissues that Gro’s repressive functions can be downregulated by EGFR/MAPK-mediated phosphorylation^47–49^; compellingly, EGFR/MAPK signaling is one of the major pathways activated upon injury and infection to promote stem cell proliferation and epithelial regeneration in the *Drosophila* midgut^37–40,50,51^. If endogenous Gro function is being downregulated by injury-induced phosphorylation, we reasoned that overexpressing Gro in injured guts might restore functional Gro levels and thus, restore lateral inhibition.

We again used the *esg*^*ts*^ driver to overexpress Gro in all progenitors of injured guts. We first examined the effect of overexpressing wild-type Gro (Gro^WT^), which is subject to the same phosphorylation-mediated downregulation as endogenous Gro. Suggestively, these tissues present milder hallmarks of damage: there are fewer multi-cell progenitor clusters and, most noticeably, reduced Delta expression in NRE-GFP:nls-expressing cells (Fig 5c). Examining single-cell NRE-GFP:nls intensities in the Delta^+^ population of injured guts with Gro^WT^ overexpression reveals a distinct decrease in Notch signaling levels, suggesting a partial reinstitution of lateral inhibition (Fig 5d). Indeed, the proportion of progenitors that are both Delta^+^ and NRE^hi^ in injured guts is reduced by ~40% with overexpression of Gro^WT^ (Fig S2d). However, ~25–55% of NRE^hi^ enteroblasts in individual guts (Fig S5c) continued to express Delta, signifying that overexpression of phosphorylation-sensitive Gro^WT^ is not able to robustly restore Notch-Delta lateral inhibition. Additionally, the wide range in proportion of Delta^+^ NRE^hi^ cells per gut suggests the degree of phosphorylation-mediated downregulation can vary between individual animals.

To account for phosphorylation-mediated downregulation of overexpressed Gro, we leveraged a Gro variant with two putative MAPK phosphorylation sites replaced by alanine residues (*gro*^*AA*^, T308A and S510A, Hasson et al 2005). We expected that this modified Gro would be resistant to phosphorylation and restore lateral inhibition more consistently than Gro^WT^. Remarkably, Gro^AA^ expression in injured guts caused them to largely resemble healthy guts, with greatly reduced abundance of esg^+^ cells, few if any progenitor clusters, and smaller, less pronounced enteroblast populations (Fig 5e). The distribution of single-cell NRE-GFP:nls intensities for Delta^+^ progenitors in injured guts with Gro^AA^ expression further supports restoration of lateral inhibition; there are far more Delta^+^ cells in the NRE^low^ population, reminiscent of homeostatic proportions (Fig 5f). Indeed, the additional presence of constitutively active Gro is enough to reduce the Delta^+^ NRE^hi^ population by ~60% (Fig S2e), such that consistently only 35–45% of injured NRE^hi^ enteroblasts still express Delta (Fig S5d). This decreased range in per gut variability compared to when we overexpressed Gro^WT^ further supports the ability of active Gro to maintain Delta repression in NRE^hi^ cells.

Taken together, these results suggest that Gro’s repressor functionality is being altered in injury, thus allowing for Delta expression to perdure in Notch-activated enteroblasts. We propose a unifying mechanism (Fig 5g) where injury-activated phosphorylation inhibits Gro, which in turn derepresses Delta downstream of Notch activation. The resulting disruption of Notch-Delta lateral inhibition is thus a direct consequence of injury-activated signals and offers an elegant means for restoring homeostatic lateral inhibition once the tissue recovers. In the absence of continued damage, mature cells no longer produce EGFR ligands, phosphorylation events are reduced, and Gro-mediated repression of Delta in enteroblasts is restored. In this way, Notch-Delta lateral inhibition can be flexibly modulated so that the tissue seamlessly switches between homeostatic and injury-responsive signaling regimes.

## Injury-induced Jak-STAT signaling is necessary and sufficient to disrupt Notch-Delta feedback

Thus far, we have explored the effects of injury on the Notch-Delta signaling circuit between progenitors. But what other tissue-wide signals coordinate injury response between the damaged mature cells and the progenitor pool tasked with rebuilding the tissue? Significant work in the field has established the conserved cytokine Jak-STAT pathway as integral to mediating regeneration and homeostasis in the *Drosophila* midgut, particularly after insults such as injury or infection^30,34,40,52–54^. Damaged and dying enterocytes release cytokines (namely Upd3) that activate Jak-STAT signaling in stem cells, stimulating increased proliferation frequency^30,52–54^. Enteroblasts also express the Jak-STAT receptor Domeless and exhibit elevated signal activation in response to injury^30,37,54^. We set out to determine how these intracellular signals affecting both stem and terminal progenitors may feed into Notch-Delta lateral inhibition regulation during injury.

**Figure 6.**
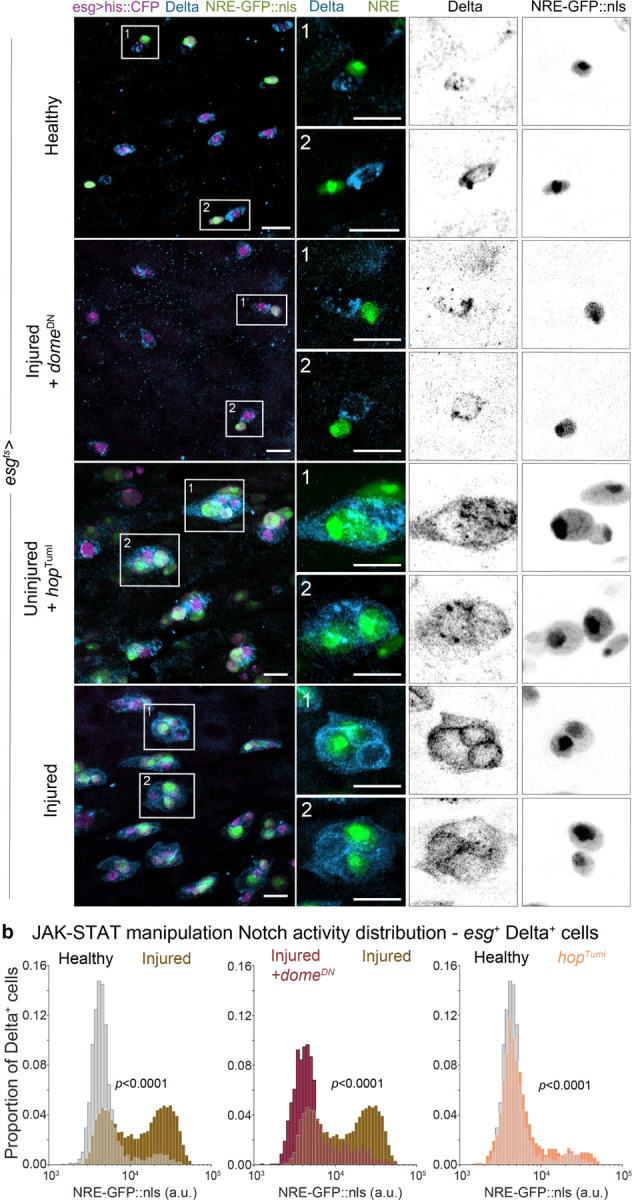
JAK-STAT is a critical mediator of injury-induced disruption of lateral inhibition. (a) Progenitor cells (*esg>his2b::CFP*, magenta) in healthy guts, injured guts with *esg*^*ts*^ driving UAS-*dome*^*DN*^ post eclosion, uninjured guts with *esg*^*ts*^*>UAS-hop*^*Tuml*^ post eclosion, and injured guts. Almost no cells expressing NRE-GFP::nls (green) also express Delta (anti-Delta immunostain, blue) in injured guts with *dome*^*DN*^ overexpression. Progenitor distribution and appearance are nearly indistinguishable from healthy despite adult day 3–4 bleomycin ingestion. Conversely, uninjured guts with *hop*^*Tuml*^ overexpression exhibit phenotypic hallmarks of injury such as large multi-cell progenitor cell clusters and strong Delta expression in NRE-GFP::nls-expressing cells. Scale bars: 10 μm. (b) Comparison of single-cell Notch signaling distributions for all Delta^+^ cells in healthy, injured + *esg*^*ts*^*>dome*^*DN*^, uninjured + *esg*^*ts*^*>hop*^*Tuml*^, and injured guts. Histograms show single-cell NRE-GFP::nls reporter intensities for all Delta^+^, *esg>his2b::CFP* cells in the gut R4ab region. The proportion of Delta^+^ NRE^low^ cells in injured guts is nearly completely restored to healthy levels by *dome*^*DN*^ overexpression. The relative proportion of Delta^+^ NRE^hi^ cells in uninjured guts is subtly but significantly increased by *hop*^*Tuml*^ overexpression. Healthy: n=3103 cells; N=19 guts. Injured +*dome*^*DN*^: n=1648 cells; N=11 guts. Uninjured +*hop*^*Tuml*^: n=2121 cells; N=11 guts. Injured: n=6690 cells; N=14 guts. *p*-values, two-sample K-S test. See also Figures S2f, S5e.

We first inhibited Jak-STAT signaling in injured guts by overexpressing a dominant-negative allele of the domeless receptor (*dome*^*DN*^) in all progenitors. In injured guts with *esg*^*ts*^*>dome*^*DN*^, Delta signal is conspicuously reduced throughout the tissue (Fig 6a), with only ~38% of all progenitors expressing Delta (Fig S2f). Analysis of single-cell Notch signaling distribution demonstrates a near-complete restoration of healthy proportions despite tissue damage, with the majority (83%) of Delta^+^ cells residing in the left, NRE^low^ peak (Fig 6b). The population of Delta^+^ NRE^hi^ progenitors is reduced from ~45% in injured guts to ~6% when Jak-STAT is blocked, matching numbers normally found in homeostatic guts (Fig S2f). Additionally, the proportion of Delta^+^ enteroblasts does not differ significantly from that of homeostatic guts (Fig S5e), indicating that injury-induced lateral inhibition disruption is completely suppressed. Taken together, these results indicate that blocking Jak-STAT signaling in injured guts restores Notch-Delta lateral inhibition, demonstrating that the injury-responsive pathway is required to lift Delta repression in Notch-activated enteroblasts.

Next, we examined the inverse case of ectopically activating Jak-STAT in uninjured guts to see if these signals are sufficient to mount an injury response and disrupt Notch-Delta lateral inhibition in the absence of damage. We overexpressed a dominant active allele of the *Drosophila* Jak kinase, hopscotch Tumorous-lethal (*hop*^*Tuml*^, H Luo 1995) in all progenitors of Delta^+^ NRE^hi^ otherwise healthy guts. Interestingly, in uninjured guts with *esg*^*ts*^*>hop*^*Tuml*^, we do see the appearance of multi-cell progenitor clusters and many large, Delta^+^, NRE-GFP:nls-expressing cells (Fig 6a). Examining single-cell NRE-GFP:nls Notch signaling distributions, the proportion of Delta^+^ NRE^hi^ progenitors increases moderately (Fig 6b); this modest relative shift is consistent with the expectation that the Delta^+^ NRE^low^ stem cell population should also proportionately increase upon Jak-STAT-activated stem cell divisions. Moreover, we quantify that the proportion of Delta^+^ NRE^hi^ lateral inhibition-disrupted progenitors is ~2x that of homeostatic guts (Fig S2f), and nearly half (~43%) of NRE^hi^ enteroblasts retain Delta expression (Fig S2f, S5e). This indicates that Jak-STAT activation in the absence of injury is indeed capable of disrupting lateral inhibition in the progenitor population.

These observations further support the notion that the relaxation of tight lateral inhibition feedback at the stem-to-terminal fate transition is an intrinsic feature of injury response. The involvement of Groucho and Jak-STAT, in conjunction with our live imaging evidence of accelerated Notch signaling during injury, describes a mechanism by which fate-determining signaling circuits can be flexibly adjusted to ramp up new mature cell generation and support rapid organ repair.

## Materials and Methods

### *Drosophila* husbandry

All experiments were performed on mated adult females. Animals were raised on standard cornmeal–molasses media (water, molasses, cornmeal, agar, yeast, Tegosept, propionic acid). For experiments, we collected adult females post-eclosion and kept them with males in cornmeal-molasses vials supplemented with a ~1cm^2^ sized dollop of yeast paste (Red Star, Active Dry Yeast mixed with water) unless otherwise noted.

Genotypes for all fixed experiments included GAL80^ts^ (i.e. *esg*^*ts*^>). We reared crosses at 18°C, collected adults on day 0 post-eclosion, then shifted flies to 29°C to inactivate GAL80^ts^ and induce GAL4-mediated expression. Flies were dissected on day 4 post-eclosion.

Live imaging experiments did not involve GAL80^ts^. Flies and crosses were kept at 25°C. We collected female flies on day 0 post-eclosion and live-imaged animals on day 3 for all conditions. During all live-imaging experiments, we fed flies via a microcapillary feeder tube with a base recipe of 10% sucrose in water.

### Bleomycin feeding to induce gut injury

To injure the gut, we fed flies Bleomycin (sulfate) (Cayman Chemical #13877) diluted in water to a final concentration of 25μg/ml and mixed into a paste with yeast (Red Star, Active Dry Yeast). For all injury experiments, we fed flies bleomycin in yeast paste as their only food source atop flugs wetted with water for 48 hours prior to dissection or live imaging. For live imaging of injured guts, we fed flies 10μg/ml bleomycin in 10% sucrose in water via a feeder tube throughout the imaging session.

### Immunostaining and sample preparation for confocal microscopy

For Ph3 staining (Fig 2d–f), guts were fixed in situ for 25–30 min at room temperature in 8% formaldehyde (Polysciences 18814–20), 200 mM sodium cacodylate, 100 mM sucrose, 40 mM KOAc, 10 mM NaOAc, and 10 mM EGTA. After fixation, guts were blocked in 0.3% PBT (0.3% Triton X-100 (Sigma-Aldrich X100) in phosphate-buffered saline (PBS)) with 5% normal goat serum (NGS; Capralogics GS0250) for 4 hours at room temperature or overnight at 4°C. Primary and secondary antibodies were incubated in 0.3% PBT + 5% NGS for 4 hours at room temperature or overnight at 4°C. Guts were washed 5 times in PBT between antibody incubations and before mounting.

For staining with mouse anti-Delta, we dissected guts into cold Schneider’s media, fixed in 4% formaldehyde in Schneider’s media at room temperature for 2 hours, and then incubated in 2N HCl in PBS for 20 minutes at room temperature. Next, we washed guts 5x 15 min with Schneider’s media and blocked in 0.3% PBT + 5% NGS at room temperature or overnight at 4°C. We incubated guts in primary antibodies in 0.3% PBT + 5% NGS for 4 hrs at room temperature or overnight at 4°C, then washed 5x 15 min in PBS before incubating with secondary antibody. Secondary antibodies were diluted in 0.3% PBT + 5% NGS, and we incubated for 4 hours at room temperature or overnight at 4°C. Finally, we again fixed guts in 4% formaldehyde in PBS for 30 min and washed 4x 15min in PBS before mounting.

We mounted immunostained guts in 3% low-melting 2-hydroxylethyl agarose (Sigma-Aldrich 39346-81-1) and Prolong Gold or Prolong Diamond Antifade mounting media (Thermo Fisher P10144, P36965). We allowed slides to dry at room temperature for 12–24 hrs and stored slides at −20°C until imaging.

We used the following primary antibodies: rabbit anti-PH3 (EMD Millipore 06–570, 1:400), mouse anti-Delta (DSHB C594–9B – concentrate 1:100, supernatant 1:20). We used the following secondary antibodies: donkey anti-mouse Alexa Fluor 647 (Invitrogen A-31571, 1:400), donkey anti-rabbit Alexa Fluor 555 (Invitrogen A-31572, 1:400). Nuclei were stained with DAPI (Invitrogen D1306, 1:1000 or 1:500).

Further details on antibodies and reagents used are provided in Supplementary Table 2.

### Confocal microscopy

Fixed samples were imaged on a Leica SP8 WLL (Fig. 2a–f) or a Leica Stellaris 8 DIVE confocal microscope with either a HC PL APO 20x immersion or a 40x oil objective (for figure images). We collected serial optical sections at 2–3μm intervals throughout the entirety of whole-mounted, immunostained guts using Leica Application Suite X (LAS X) (Version 3.5.7.23225). We used Fiji (Version 2.14.0) and Bitplane Imaris x64 (Version 10.1.1) for image analysis.

All image-based quantifications were performed on the R4ab region^55^ of the posterior midgut.

### Quantifying NRE-GFP activity distributions in fixed tissues

For all NRE-GFP::nls intensity measurements, we imaged whole-mounted guts on a Leica SP8 or Stellaris 8 DIVE confocal microscope. Initial .lif files were converted to .ims files and opened in Bitplane Imaris. We used the Add New Surfaces Function in the Surpass Module to generate surfaces for all progenitor nuclei in the *esgGAL4>his2b::CFP* (esg^+^) channel. Settings for surface recognition were kept as consistent as possible using the following settings: Smoothing enabled, Surface Grain Size = 0.5μm, Background Subtraction enabled, Diameter of Largest Sphere = 6.00μm, manual threshold value = 4400-max, region growing estimated diameter 3.60μm, ‘Classify Seed Points’ Quality adjusted for each file, ‘Classify Surfaces’ Number of Voxels adjusted for each file 10~800 voxels. Surfaces were checked for accuracy and manually edited as needed. For lateral inhibition assay experiments, we identified Delta^+^ cells via immunostaining from the existing esg^+^ surfaces and processed this Delta^+^,esg^+^ subset as a separate group. Mean NRE-GFP::nls intensity data for both Delta^+^,esg^+^ and all-esg^+^ populations was exported as .xlsx and .csv files. Files were loaded in MATLAB (R2024b) and plotted as log-scale histograms with a set bin width interval of 10^0.04^ or 10^0.05^ (Fig 2a–c). We used the two-sample Kolmogorov-Smirnov (K-S) test to evaluate statistically significant (p<0.05) difference between distributions.

Specifically for measurements of NRE-GFP::nls in PH3-stained mitotic cells (Fig 2d–f), we individually inspected PH3^+^ cells for goodness of fit to the generated surface. Surfaces that overlapped with nuclear signals from neighboring cells were edited to ensure that NRE-GFP::nls signal was only coming from the appropriate cell of interest. Cells for which an adjacent, bright GFP^+^ enteroblast interfered with accurate measurement of NRE-GFP::nls intensity were excluded from analysis.

### Analyses of NRE-GFP distributions via Gaussian Mixture Model (GMM)

Using the MATLAB fitgmdist() function, we fitted two-component Gaussian mixture models (GMMs) to the distributions of all esg^+^ progenitor cell NRE-GFP::nls intensities for each condition. We took the respective mixing proportions/prior probabilities of the two components to represent the proportions of cells residing in the NRE^low^ peak and NRE^hi^ peaks (Fig 2a–c). We took the GMM decision boundary (equal posterior probability threshold) as a proxy for the mean NRE-GFP::nls intensity where cells above this threshold are defined as NRE^hi^.

For analysis of PH3^+^ cell NRE-GFP::nls distributions (Fig 2d–f), we again fitted two-component GMMs to the distributions of all esg^+^ progenitor cell NRE-GFP::nls intensities in homeostatic and injured controls, respectively. PH3^+^ cell NRE-GFP::nls intensity distributions are displayed as raincloud plots for each condition. We computed the posterior probability prediction of each component (NRE^low^ vs NRE^hi^) for the PH3^+^ datasets against the GMM for their respective condition.

For quantification of progenitor cell Delta-Notch signaling states (Fig S2), we filtered NRE^hi^ cells from both the all esg^+^ and the Delta^+^,esg^+^ datasets for each experimental condition using the decision boundary from their respective tissue state GMM (i.e., healthy background against healthy control GMM, bleo-fed against injured control GMM), with the latter defined as the Delta^+^,NRE^hi^ group.

### Single-cell cross-correlation of Notch target and *Delta* mRNAs

We downloaded single-nuclear sequencing 10x Genomics expression matrix files for the Drosophila gut from the Fly Cell Atlas site (https://flycellatlas.org/#data) and parsed them in Python (Version 3.12.3) with Jupyter notebook. Cells from 5do female flies annotated as “intestinal stem cell” and “enteroblast” were parsed out and combined into one all-progenitor pool. We then queried all progenitors for expression levels of Delta and the three most highly expressed E(spl)-C Notch target genes (-ma, -mb, -m3, also identified in Guo et. al) as well as klumpfuss, a transcription factor induced specifically in enteroblasts (Korzelius 2019). Cells with zero levels for both Delta and the respective Notch target gene were excluded from further analysis. Normalized expression values were imported into GraphPad Prism 10 (Version 10.3.1) for plotting and correlation analysis.

### Modeling Notch-Delta lateral inhibition

We considered that the active Notch levels of a cell is an increasing function of the Delta levels of neighboring cells, and that Delta levels of the cell is a decreasing function of the active Notch levels of that cell. We formulate this interaction between pairs of cells using standard mathematical models of Notch-Delta lateral inhibition^21,28^. In its dimensionless form, the equations can be written as:

(Eq. 1) $$\frac{d{\text{N}}_{\text{1,2}}}{dt}=\frac{{\text{D}}_{2,1}^{r}}{K_{N}^{r}+{\text{D}}_{2,1}^{r}}-{\text{N}}_{\text{1,2}}$$

(Eq. 2) $$\frac{d{\text{D}}_{\text{1,2}}}{dt}=v\left(\frac{1}{1+{\left({\text{N}}_{\text{1,2}}/K_{D}\right)}^{h}}-{\text{D}}_{\text{1,2}}\right)$$

Where the subscript denotes the Notch/Delta of cell 1 or 2. In these equations, K_N_ is the dimensionless threshold of Notch activation by Delta ligand of neighboring cell, and K_D_ is the dimensionless threshold of Delta inhibition by activated Notch of the same cell. The parameter *v* is the ratio of degradation rate of Notch to Delta, which following previous work, we are assuming is equal to one^21,28,56^. According to^21^, K_N_ is inversely related to the contact area between two cells. More generally, K_N_ dictates the intercellular aspect of Notch-Delta interaction, while K_D_ dictates the intracellular aspect. The parameters r and h are the hill coefficients for Notch activation and Delta inhibition and are considered r=h=2 to account for the cooperative nature of these processes^28^.

To simulate the activation of a downstream Notch reporter, we assumed that reporter expression is directly related to activated Notch levels:

(Eq. 3) $$\frac{d{\text{Reporter}}_{1,2}}{dt}=\beta {\text{N}}_{\text{1,2}}-\alpha {\text{Reporter}}_{\text{1,2}}$$

Where *β* is the maximal production rate of reporter, and *α* is the degradation rate of reporter. Since the dimensionless Notch levels range between zero and one, the above equation would show no reporter expression prior to Notch activation and the reporter levels would reach steady state at *β*/*α* after full Notch activation. Immediately after Notch activation, the reporter expression is dominated by production rate and invariable to the degradation rate. Therefore, we approximate the reporter level by the following:

(Eq. 4) $${\text{Reporter}}_{\text{1,2}}=\int {\text{N}}_{\text{1,2}}dt$$

### Modeling simulation conditions

We numerically solved the above equations to derive the time dynamics of Notch and Delta using the odeint function from python’s scipy library. Cells are initially considered to be low Notch and high Delta. To break the symmetry between the two cells, cell 2 has a slightly higher initial Notch level than cell 1 (0.010 versus 0.011). We used a plausible range of K_N_ and K_D_ parameters to study the behavior of Notch-Delta dynamics^21,56–58^(Guisoni et al., 2017; Sprinzak et al., 2010; Pei and Baker, 2008; Friedmann and Kovall, 2010). Particularly, data fitted to wildtype cells from Guisoni et al., 2017 Figure 4^21^ shows a K_D_ range of 0.2–0.3, and a K_N_ range of 0.1–10.

### Windowmount live imaging

We performed Windowmount live imaging of the Drosophila midgut as previously described^27^. Briefly, we glued female flies to the imaging apparatus and opened a window in the dorsal cuticle of the abdomen. The R4 region of the midgut was identified, nudged through the cuticular window, and stabilized with 3% agarose before being bathed with live imaging media. We then imaged the exposed region of the midgut using an upright Leica SP5 multi-photon confocal microscope with a 20x water immersion objective (Leica HCX APO L 20x NA 1.0). We fed flies via a microcapillary feeder tube throughout the entire imaging process. Movies were captured at room temperature (20–25°C). Confocal stacks were acquired with a Z-step of 2.98 μm at 7.5min intervals and typically contained ~35–40 slices.

### Live imaging media recipe

All live imaging used the following recipe adapted from Marco Marchetti and Bruce Edgar (University of Utah), who have since published an updated version^59^: 61.5mM L-Glutamic acid monosodium salt (made in Schneider’s media), 55.5mM Trehalose (made in Schneider’s media), 2.2mM N-Acetyl Cysteine (made in water), 1.1mM Tri-sodium Citrate (made in Schneider’s media), 11% Fetal Calf Serum (or fetal bovine serum (FBS)), Schneider’s media, Penicillin-streptomycin 0.55%. Stocks of the above ingredients were made in advance, filter sterilized using a 0.2μm syringe filter, and stored at 4°C for up to 3 months. We made live imaging media fresh on the day of imaging. Media was stored at 4°C and used until the next day if needed.

### Live imaging movie registration

After acquisition, movies were processed on a Windows computer (Windows 10 Education) with a 3.70 GHz quad-core Intel Xeon processor and 128 GB memory. LIF files (*.lif) from Leica Application Suite: Advanced Fluorescence were uploaded into Fiji as a hyperstack for registration. To correct for X-Y drift, movies were converted to RGB files and processed with the Fiji plugin StackReg^60^. To correct for global volume movements, movies were processed with the Fiji plugin Correct 3D Drift^61^. We evaluated movies for viability based on criteria established in^27^.

### Live imaging cell identification, tracking, and quantification in Imaris

To perform cell tracking, processed and registered movies were converted from .tiff format to .ims file format using the Bitplane Imaris File Converter software. We performed cell segmentation in Bitplane Imaris 9.2.0 using the TransTimerRFP channel to generate 3D “spots” with the “Spots” module. All spots were generated using a standardized spot diameter of 9.02 mm. We used the Brownian motion tracking algorithm to track cell surfaces and spots for all labeled cells across all movie time points. Any errors in cell surface generation and tracking were visually inspected and corrected. Once cell recognition was verified for all cells for all time points, we exported individual cell measurements for mean intensity GFP and mean intensity RFP as Microsoft Excel files. For each channel within a movie, mean intensity values were normalized to a 0-to-1 scale by setting the maximum intensity measurement to 1. Data was imported into MATLAB or GraphPad Prism for analysis.

### Quantifying slopes of NRE>TransTimerGFP tracks

After we standardized normalizing TransTimerGFP values over time for each movie, we plotted tracks over time for each cell and smoothed the data using the ‘rlowess’ method and a moving time-average spanning 5 timepoints in MATLAB. Cells were excluded from further analysis if the average of the first half of the data points in the track were <0.1 mean GFP intensity. Cells that still had visible TransTimerRFP expression but had TransTimerGFP intensity < 0.1 were designated as recently Notch-OFF cells that were excluded from slope analysis. Next, to enable accurate slope analysis of tracks with distinct positive and negative slope segments, we split tracks into two parts at the maximum value of the smoothed data. Data before the maximum should have a positive slope, and after, a negative slope. We then fit the equation (y=mx+b) to the smoothed data. Fitted lines were excluded from further analysis if: (1) there were fewer than 8 data points for the line to fit or (2) the slope of the fit line had an opposite direction (+ or −) slope from what it should. Slope measurements were separated into positive and negative slopes for plotting and comparison.

### Statistical analyses

Statistical analyses and histogram plotting for fixed NRE-GFP::nls quantifications were done in MATLAB and edited in Adobe Illustrator (Version 29.0). For comparisons of NRE-GFP::nls distributions, we used the two-sample Kolmogorov-Smirnoff (K-S) test to assess statistical significance.

All plots for TransTimer tracks and slopes (Fig 4g, i–l), single-cell cross correlation plots (Fig S3), and violin plots of Delta^+^,NRE^hi^ proportions (Fig S5) were made in GraphPad Prism 10 and edited in Adobe Illustrator. For comparisons of distributions of cell slopes, we used unpaired two-tailed Mann-Whitney tests to assess median and statistical significance. For comparisons of cell numbers, we used unpaired Student’s two-tailed t-tests to assess mean and statistical significance. For single-cell cross-correlation (Fig S3), we used Pearson correlation coefficients (r) and p-values (two-tailed t-test) to assess correlation and statistical significance. For Delta^+^,NRE^hi^ violin plots (Fig S4), we used ordinary one-way ANOVA with Tukey’s multiple comparisons test to assess mean and statistical significance.

The number of experimental replicates for each assay is indicated in the figure legends. Statistical tests used are indicated in the figure legends.

For all experiments, randomization was not relevant/not performed. Data collection and analysis were not performed blind to the conditions of the experiments. All data were acquired and processed identically and in parallel. We used GraphPad Prism 8/9/10 (Versions 8.0.0 through 10.3.1), Microsoft Excel 365 (Version 16.90), MATLAB (R2024b), and Python (Version 3.12.3) for statistics and graph generation. We used Adobe Illustrator (Version 29.0) for figure assembly.

## Supplementary Material

Supplement 1

Supplement 2

Supplement 3

Supplement 4

Supplement 5

Supplement 6

Supplement 7

1

## Figures and Tables

**Table 1 –:** Genotypes in Figure Panels

FIGURE	GENOTYPE
Fig 1c	esgGAL4, UAS-his2b::CFP, GBE-Su(H)-GFP:nls/+; ubi-E-cadherin::YFP/+
Fig 2a–k	w1118; esgGAL4, UAS-his2b::CFP, GBE-Su(H)-GFP:nls/+; tubGAL80ts/+
Fig 4e–l	NRE>TransTimer: GBE-Su(H)GAL4/Cyo; UAS-IVS-syn21-nls-sfGFP-MODC-P2A-nlsTagRFP(attP2)/TM3,Ser
Fig 5a,b	w1118; esgGAL4, UAS-his2b::CFP, GBE-Su(H)-GFP:nls/+; tubGAL80ts/+, esgGAL4, UAS-his2b::CFP, GBE-Su(H)-GFP:nls/+; tubGAL80ts/UAS-groRNAi^KK110546^, esgGAL4, UAS-his2b::CFP, GBE-Su(H)-GFP:nls/UAS-groRNAi^BL91407^; tubGAL80ts/+
Fig 5c,d	esgGAL4, UAS-his2b::CFP, GBE-Su(H)-GFP:nls/UAS-Gro.CC; tubGAL80ts/+
Fig 5e,f	esgGAL4, UAS-his2b::CFP, GBE-Su(H)-GFP:nls/UAS-Gro.AA; tubGAL80ts/+
Fig 6a,b	w1118; esgGAL4, UAS-his2b::CFP, GBE-Su(H)-GFP:nls/+; tubGAL80ts/+, esgGAL4, UAS-his2b::CFP, GBE-Su(H)-GFP:nls/UAS-dome Δcyt 3–1; tubGAL80ts/Dr UAS-hopTuMl; esgGAL4, UAS-his2b::CFP, GBE-Su(H)-GFP:nls/+; tubGAL80ts/+,

**Table 2 –:** Reagents and Resources

REAGENT or RESOURCE	SOURCE	IDENTIFIER
Antibodies		
Mouse anti-Delta (concentrate 1:100, supernatant 1:20)	DSHB	C594–9B
Mouse anti-Phospho-histone H3 (1:400)	EMD Millipore	06–570
Donkey anti-mouse Alexa Fluor 647	Invitrogen	A-31571; RRID: AB_162542
Donkey anti-rabbit Alexa Fluor 555	Invitrogen	A-31572; RRID: AB_162543
Chemicals, Peptides, and Recombinant Proteins		
Bleomycin (sulfate) (25μg/ml)	Cayman Chemical	13877; CAS Number 9041-93-4
DAPI (1:1000)	Invitrogen	D1306
Prolong Gold antifade	Thermo Fisher	P10144
Prolong Diamond antifade	Thermo Fisher	P36970
Gibco^™^ Schneider’s Drosophila Medium	Thermo-Fisher Scientific	21720024
L-Glutamic acid monosodium salt	Spectrum Chemical MFG Corp.	GL135–500GM; CAS: 6106-04-3
D-(+)-Trehalose	Sigma-Aldrich	IT9449–25G; CAS:6138-23-4
N-Acetyl Cysteine	Cayman Chemical Company	20261; CAS:616-91-1
Tri-sodium Citrate	Sigma-Aldrich	PHR1416–1G; CAS:6132-04-3
Fetal Bovine Serum	Sigma-Aldrich	F4135–100ML
Penicillin-streptomycin	Thermo Fisher	BW17–745H
Sodium Cacodylate	Sigma-Aldrich	C0250–25G; CAS: 6131-9-3
Formaldehyde	Polysciences	18814–20
Sucrose	Sigma-Aldrich	84097–250G; CAS: 57-50-1
KOAc	Sigma-Aldrich	P1190–100G; CAS:127-08-2
NaOAc	Sigma-Aldrich	S2889–250G; CAS:127-09-3
EGTA, for molecular biology ≥ 97%	Sigma-Aldrich	E3889; CAS: 67-42-5
2-hydroxyethylagarose	Sigma-Aldrich	A4018; CAS: 39346-1-1
KWIK-SIL adhesive silicon glue	World Precision Instruments	KWIK-SIL
Experimental Models: Organisms/Strains					
*Drosophila: w; ubi-E-cadherin::YFP; + --*	Denise Montell	PMID: 24855950
*Drosophila: GBE-Su(H)-GFP:nls* ;+	Joaquin de Navascués lab	PMID: 22522699
*Drosophila: esg-GAL4; +*	Kyoto DGGR	112304; FLYB: FBti0033872
*Drosophila: UAS-his2b::CFP*	Yoshihiro Inoue lab (Miyauchi et al. 2013)	PMID: 24850412
Drosophila: w[*]; P{w[+mC]=tubP-GAL80[ts]}2/TM2	BDSC	7017; FLYB: FBti0027797
*Drosophila: esgGAL4, UAS-his2b::CFP, GBE-Su(H)-GFP:nls/Cyo; tubGAL 80ts/(TM6B, Tb,Hu)*	This paper	esg^ts^, NRE
*Drosophila: w1118; +; +*	BDSC	RRID: BDSC_5905
*Drosophila: UAS-groRNAi (#1)*	VDRC	KK110546
Drosophila: y[1] sc[*] v[1] sev[21]; P{y[+t7.7] v[+t1.8]=TRiP.HMS06033}attP40/CyO (UAS-groRNAi #2)	BDSC	RRID: BDSC_91407
*Drosophila: UAS-groORF-CC; +/+* (Gro^WT^)	FlyORF	FBgn0001139
Drosophila: w[*]; P{w[+mC]=UAS-gro.AA}2/CyO (Gro^AA^)	BDSC	RRID: BDSC_76323
*Drosophila: UAS-hopTuml; +; +*	David Bilder lab	
*Drosophila: w; UAS-dome [Delta]cyt 3–1/Cyo; Dr/TB6C*	David Bilder lab	
*Drosophila: GBE-Su(H)-GAL4; + (NRE>)*	Steve Hou	
*Drosophila: If/Cyo; UAS-IVS-syn21-nls-sfGFP-MODC-P2A-nlsTagRFP(attP2) (UA S-TransTimer)*	Norbert Perrimon	
*Drosophila: GBE-Su(H)-GAL4/Cyo; UAS-IVS-syn21-nls-sfGFP-MODC-P2A-nlsTagRFP(attP2)/TM3, Ser (NRE> TransTimer)*	This paper	

## Data Availability

All data and code that support the findings of this study are available from the authors upon reasonable request.
