# Supplementary material for: Organ injury accelerates stem cell differentiation by modulating a fate-transducing lateral inhibition circuit
Source: bioRxiv. 2024 Dec 30:2024.12.29.630675. Preprint. [Version 1] doi: 10.1101/2024.12.29.630675 (PMC11722240; doi:10.1101/2024.12.29.630675)
Supplement: Supplementary file 1 [file NIHPP2024.12.29.630675V1-supplement-1.pdf]

bioRxiv preprint doi: https://doi.org/10.1101/2024.12.29.630675; this version posted December 30, 2024. The copyright holder for this preprint (which was not certified by peer review) is the author/funder, who has granted bioRxiv a license to display the preprint in perpetuity. It is made available under aCC-BY 4.0 International license.

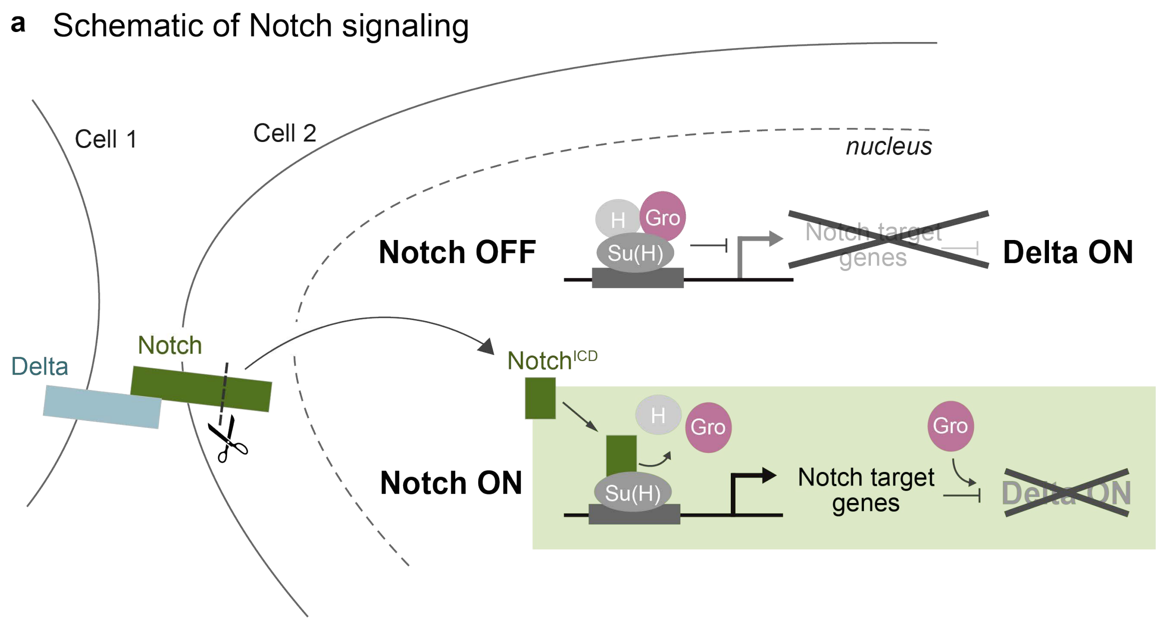

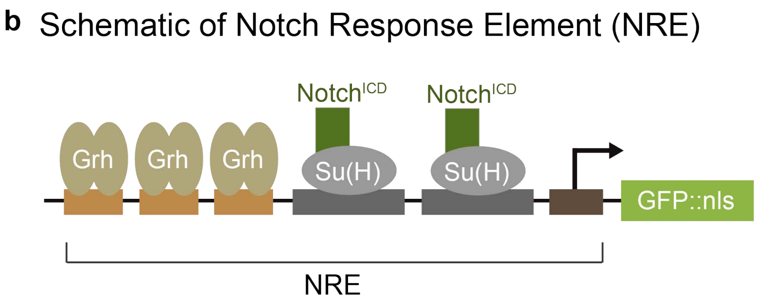

Supplemental Figure 1: Molecular regulation of Notch target genes and the Notch Response Element (NRE)

(a) Simplified schematic of Notch target regulation. In the inactive state (Notch OFF), Suppressor of Hairless (Su(H)) bound to DNA sites (gray boxes) recruits co-repressors Hairless (H) and Groucho (Gro), silencing Notch targets while permitting Delta expression. In the active state, Delta ligand (blue) binds Notch receptor (green) (Notch ON), releasing the Notch intracellular domain ($Notch^{ICD}$). $Notch^{ICD}$ enters the nucleus, binds Su(H), and displaces H/Gro. The $Notch^{ICD}$/Su(H) complex then drives Notch target gene expression. Notch targets, together with Gro, repress Delta transcription.

(b) Structure of the Notch Response Element (NRE) reporter. Sensitive detection of Notch activation is conferred by the combination of two Su(H) binding sites with three binding sites for the transcriptional activator Grainyhead (Grh) (Furriols and Bray, 2001). The NRE drives expression of nuclear GFP (GFP:nls) in all figures except Figure 4, where it drives GAL4.

bioRxiv preprint doi: https://doi.org/10.1101/2024.12.29.630675; this version posted December 30, 2024. The copyright holder for this preprint (which was not certified by peer review) is the author/funder, who has granted bioRxiv a license to display the preprint in perpetuity. It is made available under aCC-BY 4.0 International license.

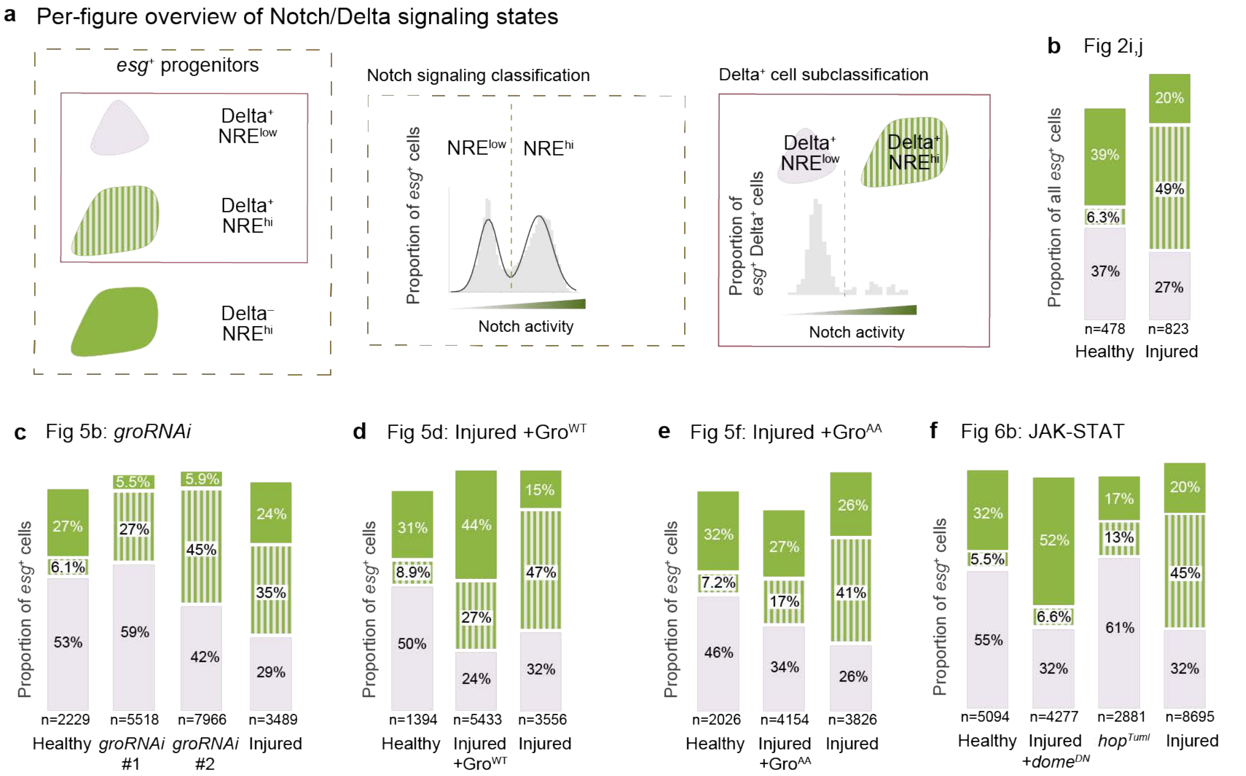

Supplemental Figure 2: Overview of Notch/Delta signaling states across experimental conditions

(a) Classification framework for Notch/Delta signaling states in midgut progenitors (*esg*$^+$). (b-f) Quantitation of signaling states (percent of total *esg*$^+$ cells) for: (b) Fig 2i,j: Healthy vs injury; (c) Fig 5b: *gro* RNAi; (d) Fig 5d: Injury + *gro*^{WT}; (e) Fig 5f: Injury + *gro*^{AA}; (f) Fig 6b: JAK-STAT pathway perturbations. Values shown as percentage of total *esg*$^+$ cells. Delta$^-$, NRE^{low} cells excluded as they do not signal, so proportions sum to <100%.

bioRxiv preprint doi: https://doi.org/10.1101/2024.12.29.630675; this version posted December 30, 2024. The copyright holder for this preprint (which was not certified by peer review) is the author/funder, who has granted bioRxiv a license to display the preprint in perpetuity. It is made available under aCC-BY 4.0 International license.

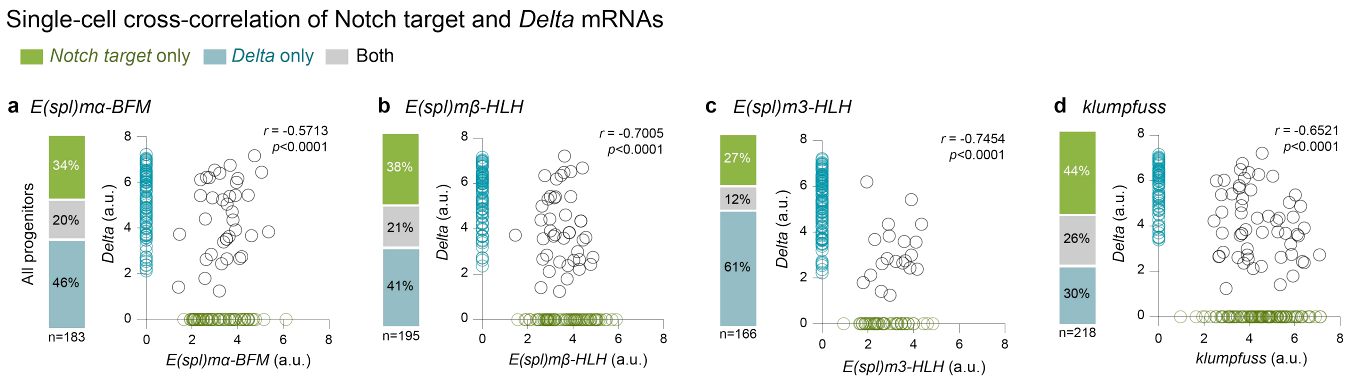

Supplemental Figure 3: Anti-correlation of Delta and Notch target mRNAs in healthy-gut progenitors.

Single-cell expression analysis of Delta and four major midgut Notch target genes: (a) E(spl)mα-BFM, (b) E(spl)mβ-HLH, (c) E(spl)m3-HLH, and (d) klumpfuss (Guo 2019, Bardin 2010, Korzelius 2019). Stacked bars quantify proportions of progenitor cells expressing Delta-only (blue), Notch target-only (green) or both (gray). Scatter plots show Delta versus Notch target mRNA levels per cell, with corresponding color-coding. Data from 5-day-old, mated female flies (Fly Cell Atlas, Li et al. 2022). See Methods. r = Pearson's correlation coefficient; *p*-values from two-tailed t-test.

bioRxiv preprint doi: https://doi.org/10.1101/2024.12.29.630675; this version posted December 30, 2024. The copyright holder for this preprint (which was not certified by peer review) is the author/funder, who has granted bioRxiv a license to display the preprint in perpetuity. It is made available under aCC-BY 4.0 International license.

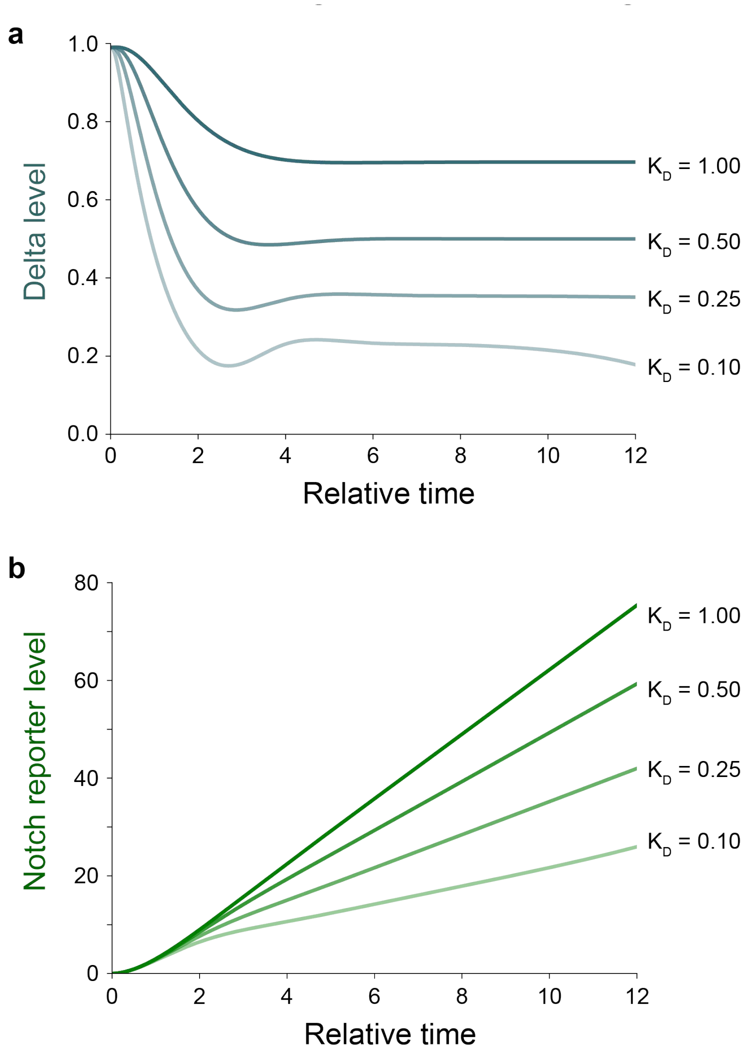

Supplemental Figure 4: Delta and Notch signaling dynamics across K_D values

Simulated time evolution of (a) Delta levels and (b) Notch reporter levels at the indicated K_D values. Increased K_D produces higher levels of both Delta and Notch reporter. K_N =0.5 in all simulations.

bioRxiv preprint doi: https://doi.org/10.1101/2024.12.29.630675; this version posted December 30, 2024. The copyright holder for this preprint (which was not certified by peer review) is the author/funder, who has granted bioRxiv a license to display the preprint in perpetuity. It is made available under aCC-BY 4.0 International license.

Movie 1: 20-hour live imaging movie of a healthy NRE>TransTimer gut
See Figure 4e. Two-channel, wide-field, volumetric movie of a healthy NRE>TransTimer gut. White lines initially outline the gut boundaries. NRE>TransTimerGFP (green) marks cells with active Notch signaling. NRE>TransTimerRFP (magenta) marks recent Notch signaling activity. Scale bar, 50µm.

Movie 2: 20-hour live imaging movie of an injured NRE>TransTimer gut
See Figure 4f. Two-channel, wide-field, volumetric movie of an injured NRE>TransTimer gut. White lines initially outline the gut boundaries. NRE>TransTimerGFP (green) marks cells with active Notch signaling. NRE>TransTimerRFP (magenta) marks recent Notch signaling activity. Scale bar, 50µm.

Movie 3: Healthy NRE>TransTimer cell exhibiting NRE activation
See Figure 4g, Cell 1. Cell in frame increases both NRE>TransTimerGFP (first panel, green; second panel, inverted gray) and NRE>TransTimerRFP (first panel, magenta; third panel, inverted gray) signal over the course of the 20-hour movie. Each time point is the projection of a confocal z-stack. Scale bar, 5µm.

Movie 4: Healthy NRE>TransTimer cell exhibiting stable NRE signal
See Figure 4g, Cell 2. The centermost GFP+ cell in frame exhibits stable NRE>TransTimerGFP (first panel, green; second panel, inverted gray) and NRE>TransTimerRFP (first panel, magenta; third panel, inverted gray) signal over the course of the 20-hour movie. Each time point is the projection of a confocal z-stack. Scale bar, 5µm.

Movie 5: Healthy NRE>TransTimer cell exhibiting NRE deactivation.
See Figure 4g, Cell 3. The centermost GFP+ cell in frame (denoted by white arrow) exhibits decreasing NRE>TransTimerGFP (first panel, green; second panel, inverted gray) and NRE>TransTimerRFP (first panel, magenta; third panel, inverted gray) signal over the course of the 20-hour movie. Each time point is the projection of a confocal z-stack. Scale bar, 5µm.

Movie 6: Injured NRE>TransTimer cell exhibiting both NRE activation and deactivation.
See Figure 4h. Cell in frame exhibits both increasing and decreasing NRE>TransTimerGFP (first panel, green; second panel, inverted gray) and NRE>TransTimerRFP (first panel, magenta; third panel, inverted gray) signal in the course of the 20-hour movie. Each time point is the projection of a confocal z-stack. Scale bar, 5µm.

bioRxiv preprint doi: https://doi.org/10.1101/2024.12.29.630675; this version posted December 30, 2024. The copyright holder for this preprint (which was not certified by peer review) is the author/funder, who has granted bioRxiv a license to display the preprint in perpetuity. It is made available under aCC-BY 4.0 International license.

Proportion of Delta^{+} NRE^{hi} cells

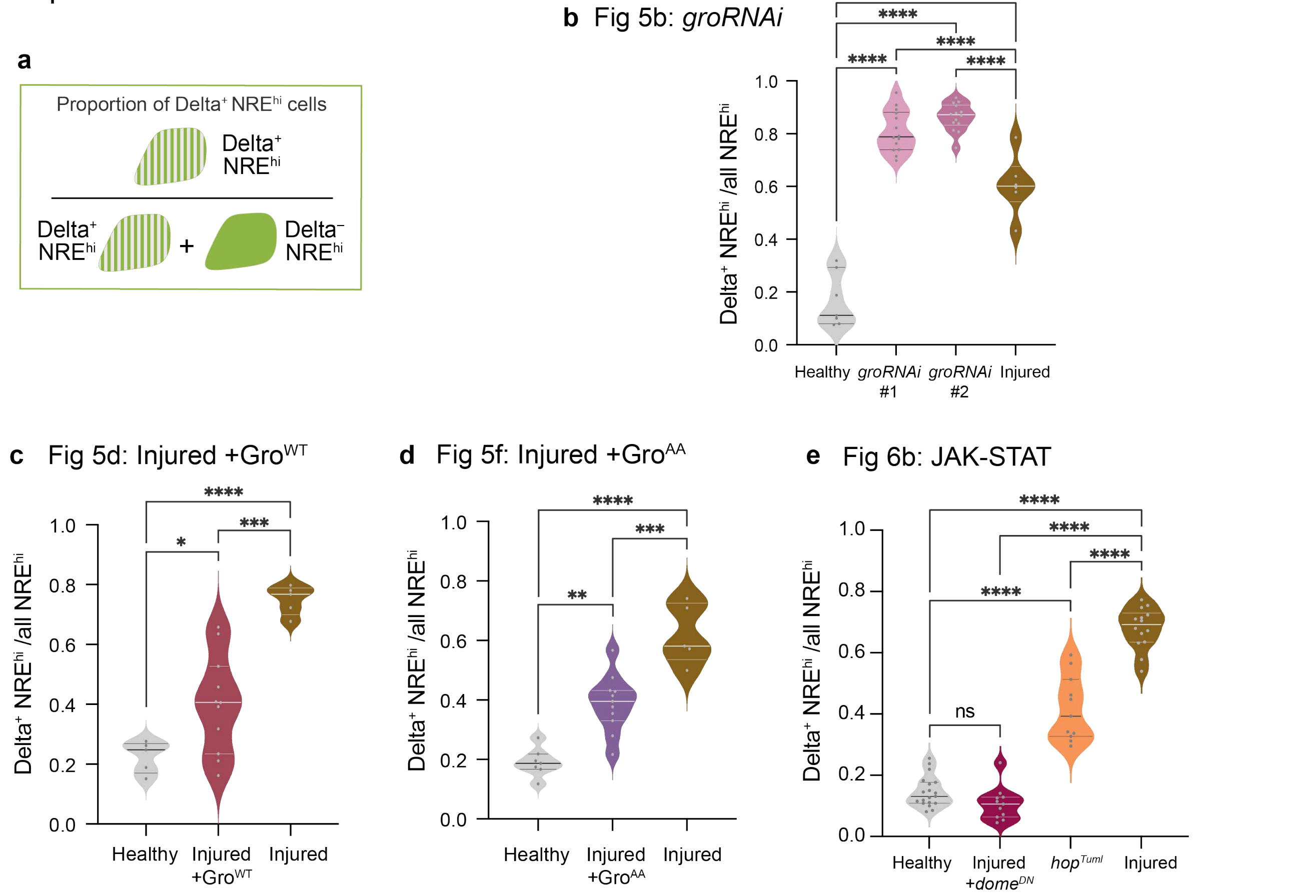

Supplemental Figure 5: Analysis of the proportion of Delta^{+} NRE^{hi} enteroblasts on a per gut basis across conditions

(a) Schematic of calculation for proportion of NRE^{hi} cells that are Delta^{+}. Violin plots of the proportion of NRE^{hi} cells that are Delta^{+} for data corresponding to: (b) Fig 5b, (c) Fig 5d, (d) Fig 5f, and (e) Fig 6b. Each dot represents one gut. Horizontal lines represent median and 25th, 75th percentiles. *p*-values, Ordinary one-way ANOVA with Tukey's multiple comparisons. ns, not significant; *, p<0.05; **, p<0.01; ***, p <0.001; ****, p<0.0001.
