# Supplementary material for: Organ injury accelerates stem cell differentiation by modulating a fate-transducing lateral inhibition circuit
Source: bioRxiv. 2024 Dec 30:2024.12.29.630675. Preprint. [Version 1] doi: 10.1101/2024.12.29.630675 (PMC11722240; doi:10.1101/2024.12.29.630675)
Supplement: Supplementary file 2 [file media-1.pdf]

Three-cell Model of ISC Notch Delta Signaling

Saman Tabatabaee

October 2024

1 Introduction

Upon injury, Drosophila intestinal cells exhibit a unique Delta-positive Notch-positive population. Previous work using a two-cell model of Notch Delta signaling adapted from Guisoni et al. 2017 was sufficient to recreate this observation. More specifically, disruption of the inhibitory loop between Notch and Delta was necessary to create this effect, and furthermore, this perturbation is predicted to increase Notch activation speed, which was experimentally proven to be true.

It was also observed that upon injury, there is an increased instance of multiple cells contacting each other, suggesting that the two-cell model is no longer accurate. Additionally, it was suggested that the emergence of the Delta-positive Notch-positive population may be due to this clustering effect, and not by the disruption of Delta inhibition as seen in the two-cell model. To address these concerns, I have generated a three-cell model of Notch Delta signaling to explore whether the addition of another cell to the model is sufficient in generating a high-Delta high-Notch state.

2 Model 1

To generate a three-cell model, I decided to extend the two cell model based on previous lattice models of Notch-Delta signaling (Sprinzak et al. 2010). Briefly, the two-cell model from Guisoni et al. 2017 in its reduced form has the following equations:

$$\frac{\mathrm{d}N_{1,2}}{\mathrm{d}t} = \frac{D_{2,1}^r}{K_N^r + D_{2,1}^r} - N_{1,2} \tag{1}$$

$$\frac{\mathrm{d}D_{1,2}}{\mathrm{d}t} = v\left(\frac{1}{1 + \left(N_{1,2}/K_D\right)^h} - D_{1,2}\right) \tag{2}$$

Where v is the ratio of Notch and Delta degradation rates and generally assumed to equal to one. The main parameters in this model are K_N and K_D which are respectively the threshold of Notch activation by neighboring Delta, and Delta inhibition by Notch of the same cell. According to Guisoni et al. 2017, K_N is inversely related to cell-cell contact area, in other words, cells with high contact area would have more Notch receptors getting activated by neighboring Delta, and thus lower K_N. Conversely, cells with little contact area would not have any Notch activation, and thus K_N would be large.

In the literature, modeling multi-cell Notch-Delta signaling makes the assumption that cells are on a lattice and all cells have similar surface area contacts. Therefore, the only variables that become important are the Notch and Delta levels of neighboring cell. The Sprinzak et al. 2010 model is based on these assumptions and it takes the following form:

$$\frac{\mathrm{d}N_i}{\mathrm{d}t} = \beta_N - \gamma_N \cdot N_i - \kappa_t^{-1} N_i \langle D_i \rangle \tag{3}$$

$$\frac{\mathrm{d}R_i}{\mathrm{d}t} = \beta_R \frac{\left(\frac{\kappa_t^{-1}}{\gamma_S} N_i \langle D_i \rangle\right)^m}{p_s^m + \left(\frac{\kappa_t^{-1}}{\gamma_S} N_i \langle D_i \rangle\right)^m} - \gamma_R \cdot R_i \tag{4}$$

$$\frac{\mathrm{d}D_i}{\mathrm{d}t} = \beta_D \frac{p_R^l}{p_R^l + R_i^l} - \gamma_D D_i - \kappa_t^{-1} D_i \langle N_i \rangle \tag{5}$$

In these equations, there are three variables: Notch, Delta, and downstream repressor. Notch activates a downstream repressor which then represses Delta. As a result, there is a threshold for repressor activation p_s, and a threshold for Delta inhibition p_R. Additionally, β is the rate of protein synthesis and γ is the rate of protein degradation. Unlike the previous model where Notch activation is dictated by K_N, here Notch activation is dictated by the interaction of Notch with $\langle D \rangle$, which is the sum of neighboring Delta levels, and κ_t^{-1} denotes the strength of transactivation. For more detail, please read the review by Binshtok and Sprinzak 2018.

To extend the Guisoni et al. model to three cells, we can take inspiration by this notation to generate the following equations:

$$\frac{\mathrm{d}N_i}{\mathrm{d}t} = \frac{\langle D \rangle_i^r}{K_{N_i}^r + \langle D \rangle_i^r} - N_i \tag{6}$$

$$\frac{\mathrm{d}D_i}{\mathrm{d}t} = \frac{1}{1 + (N_i/K_D)^h} - D_i \tag{7}$$

Since the intracellular process of Delta inhibition should not theoretically change in the presence of more cells, the equation governing Delta dynamics remains the same as before. In equation (6), $\langle D \rangle$ is the sum of Delta levels of neighboring cells, and K_{N_i} is the threshold for Notch activation for cell i, which is inversely related to the combined contact area of cell i with neighboring cells.

While this model replicates the structure of the Sprinzak multi-cell model, it is flawed. This can be shown with the extreme case where cell 1 and 2 are contacting each other while cell 3 is not in contact with either. In this example, the three-cell model should reduce down to the two cell model, but in the above formulation, the Delta levels of cell 3 would still be included in the equation 6 and affect the outcome of cell 1 activation, while the reduced contact area affects K_N in a non-linear fashion and complicate the proper cell 1-2 activation.

3 Model 2

To avoid the failure of Model 1, we need to change the Hill function that describes Notch activation in equation 6. If cell 2 is making large contact with cell 1, it should be sufficient to activate cell 1 independent of cell 3 contact area. If cell 2 is activating few receptors independently and cell 3 is activating few receptors, this effect should be additive. This means that if cell 2 and 3 have the same number of Delta ligands, their combined effect would be the same as cell 2 making twice the amount of contact alone. In other words, Notch activation of cell 1 require an OR logic, where activation from either cell 2 or 3 is sufficient, and the combined effect should also be additive.

To employ this OR logic, we take inspiration from Guisoni et al. 2017's definition of $K_N = c/A$, where c is a constant and A is the area of contact. By plugging this definition back into equation (1), we get the following activation function formulation:

$$\frac{(A \cdot D)^r}{c^r + (A \cdot D)^r} \tag{8}$$

To turn this equation to account for two pairs of interactions, we can then write it as the following:

$$\frac{(A_{1-2}D_2 + A_{1-3}D_3)^r}{c^r + (A_{1-2}D_2 + A_{1-3}D_3)^r} \tag{9}$$

Where A_{1-2} is the contact area between cell 1 and 2. In this formulation, if cell 3 is not contacting cell 1, then $A_{1-3}D_3$ would be zero and thus remove the cell 3 Delta contribution. The constant c is the same as in the 2 cell model, and thus the only addition would be the Notch activation from the additional cell. This way of introducing OR logic into a Hill function is also used in other studies (Kirouac et al. 2013), and we feel that it appropriately encompasses the contribution of additional cells. The general Notch-Delta dynamics can be written as the following:

$$\frac{\mathrm{d}N_i}{\mathrm{d}t} = \frac{\left(\sum_{i \neq j} A_{i-j} D_j\right)^r}{c^r + \left(\sum_{i \neq j} A_{i-j} D_j\right)^r} - N_i \tag{10}$$

$$\frac{\mathrm{d}D_{1,2}}{\mathrm{d}t} = v\left(\frac{1}{1 + (N_{1,2}/K_D)^h} - D_{1,2}\right) \tag{11}$$

The parameter c is a constant that can be assumed to be one and v is also assumed to be equal to one in the literature. With these assumptions, the model has four total parameters: K_D which should be theoretically the same between different cells, and three surface area variables A_{1-2}, A_{2-3}, and A_{3-1}.

4 Simulation

4.1 Three back-to-back cells

The first situation that we considered is one where three cells are in contact in an end-to-end orientation. If cell 1 is the middle cell,then there is no contact between cell 2 and 3, then the parameter A_{2-3} is set to zero. Using this simplification, we can assess the effect of additional cell contact on Notch and Delta levels at equilibrium. To keep these examples comparable to the two-cell model, we calculated the equilibrium levels as a function of K_D and A_{1-2} while keeping A_{1-3} fixed (Figure 1). In these examples, setting the A_{1-3} to zero reproduces the two cell model, and in this limit, $1/A_{1-2}$ is the same as K_N in the two-cell model plots.

What becomes clear in these simulations is that with increasing cell contact of an additional cell (cell 3), the Delta dynamics become less and less dependent on cell 2 contact. For example, at A_{1-3} of 100, cell 1 is going to differentiate as shown by the high Notch state for all variables of A_{1-2}. Additionally, since cell 1 is in contact with both cell 2 and 3, there is an asymmetric regime where cell 1 differentiates while cell 2 remains stem as shown by high-Delta low-Notch (Figure 2). Cell 3 dynamics are largely independent of cell 2 dynamics since they are not directly contacting one another (Figure 3). In cell 3, if A_{1-3} is very low, the cell stays as stem, and if A_{1-3} is very high, the cell will generally differentiate except for a low K_D regime. The only dependence between cell 2 and cell 3 occurs at $A_{1-3} \approx 1$, where the combined effects of cell 2 and 3 push cell 1 to differentiate.

When looking at Delta levels across all three cells, except for the asymmetric regime between cell 1 and 2, the only way to increase equilibrium Delta levels is by increasing K_D, which is what the two-cell model also suggests. These simulations show that for the range of parameters tested, increasing the number of cells in the model is not going to induce a high-Delta high-Notch state without an increase in K_D. Mathematically speaking, the addition of cells is incorporated via their contact area in the Notch dynamics equation, and the addition of contacting cells is another way of changing K_N of the two-cell model. And as it was shown previously, changing K_N alone cannot reproduce the high-Delta high-Notch state.

Another way of showing the same effect is to keep K_D constant and look at the effect of A_{1-2} and A_{1-3} on Notch and Delta levels (Figure 4). In general, for a low K_D value (Figure 4a), a high-Delta high-Notch state is not achievable, while for higher K_D values (Figure 4b-c), not only a high-Delta high-Notch state becomes achievable, a low-Delta high-Notch state is no longer feasible.

From the two-cell model results, we predicted that the increase in K_D would accompany an increase in Notch activation speed. We wondered whether the cell clustering had an effect on this. To assess this, we generated the Notch activation speed for cell 1,2, and 3 as a function of K_D and A_{1-2} while keeping A_{1-3} fixed (Figure 5). Quite interestingly, the Delta and Notch equilibrium levels of cell 1 are not affected at low A_{1-3} values of 0.001-0.01 (Figure 1), but Notch activation speed increases in this regime (Figure 5a). This effect can also be shown by fixing K_D and looking at Notch activation speed as a function of A_{1-2} and A_{1-3} (Figure 6). This effect is more clear for K_D of 0.1 and 0.5, before Notch activation speed reaches maximal level due to the increasing K_D.

4.2 Conclusions

From studying the model of a simple multi-cell interaction where three cells are interacting in an end-to-end fashion, we saw that increasing cell-cell contact cannot generate a high-Delta high-Notch state similar to the

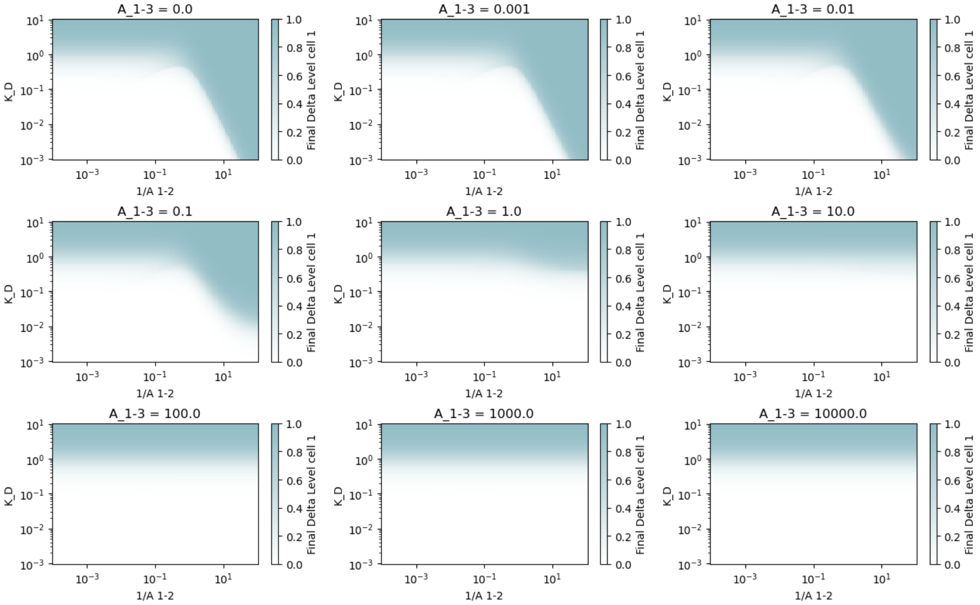

(a)

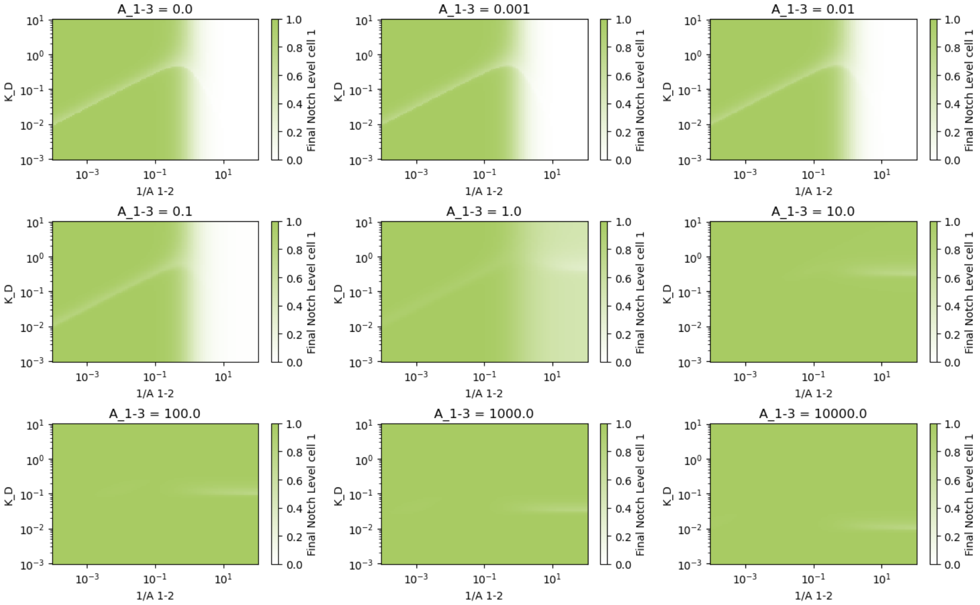

(b)

Figure 1: Equilibrium level of A) Delta and B) Notch as a function of K_D and A_{1-2} while holding A_{1-3} fixed for the cell 1 (the middle cell).

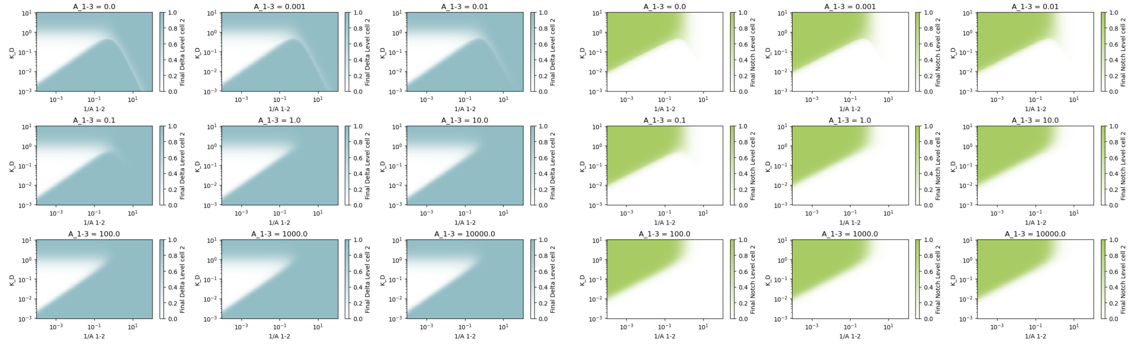

(a) (b)

Figure 2: Equilibrium level of A) Delta and B) Notch as a function of K_D and A_{1-2} while holding A_{1-3} fixed for the cell 2 (one of the side cells).

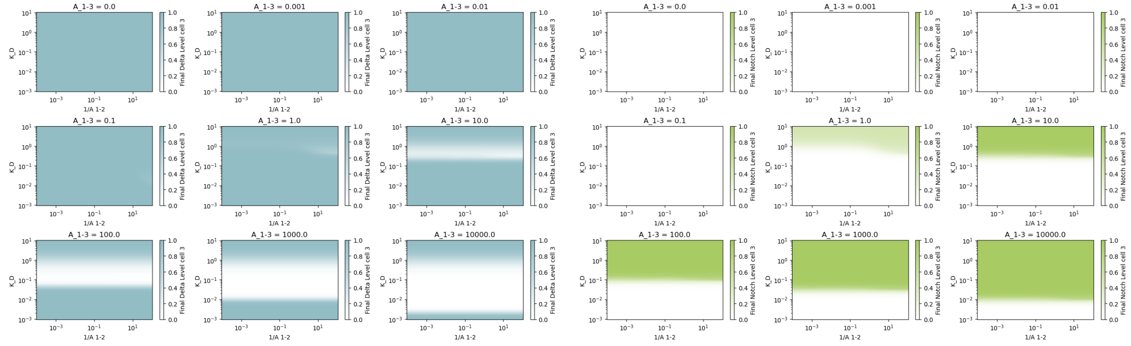

(a) (b)

Figure 3: Equilibrium level of A) Delta and B) Notch as a function of K_D and A_{1-2} while holding A_{1-3} fixed for the cell 3 (the other side cell).

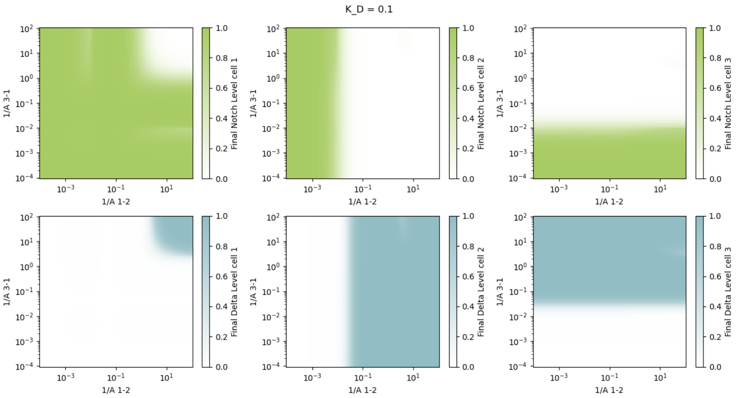

(a)

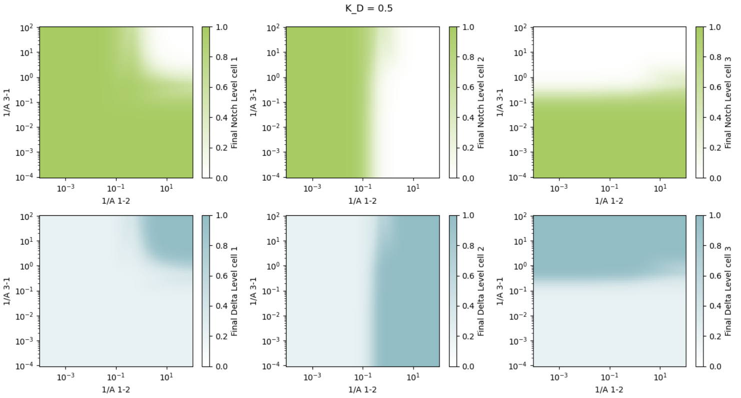

(b)

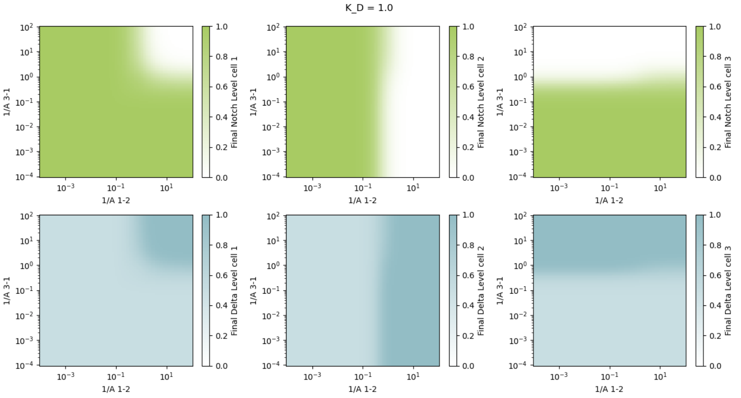

(c)

Figure 4: Equilibrium level of Notch and Delta as a function of A_{1-2} and A_{1-3} for a fixed K_D of a) 0.1, b) 0.5, c) 1.0.

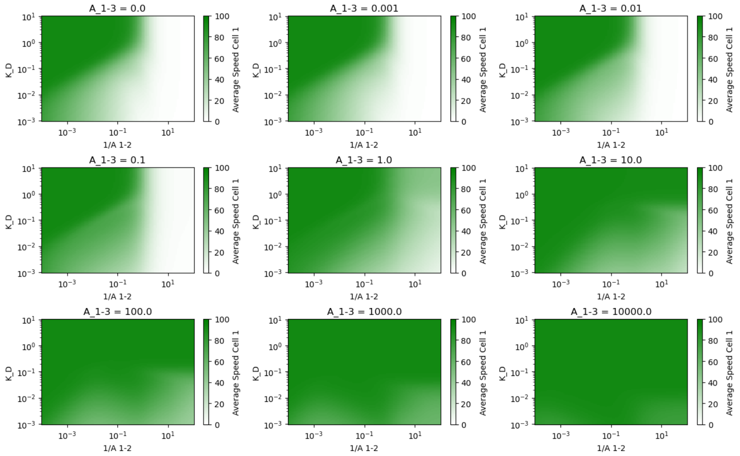

(a)

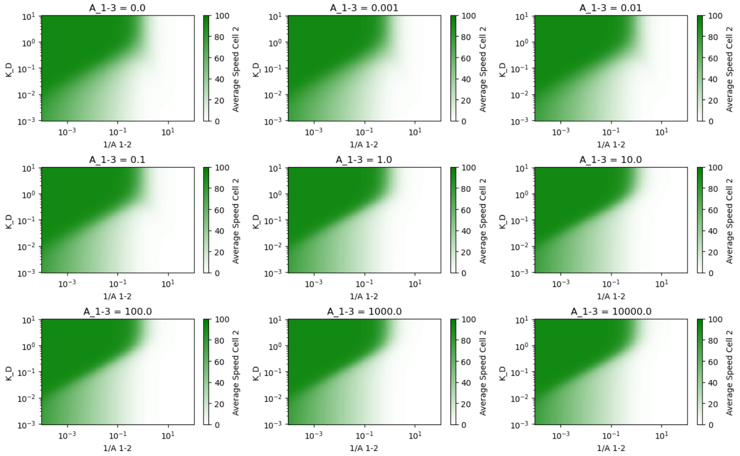

(b)

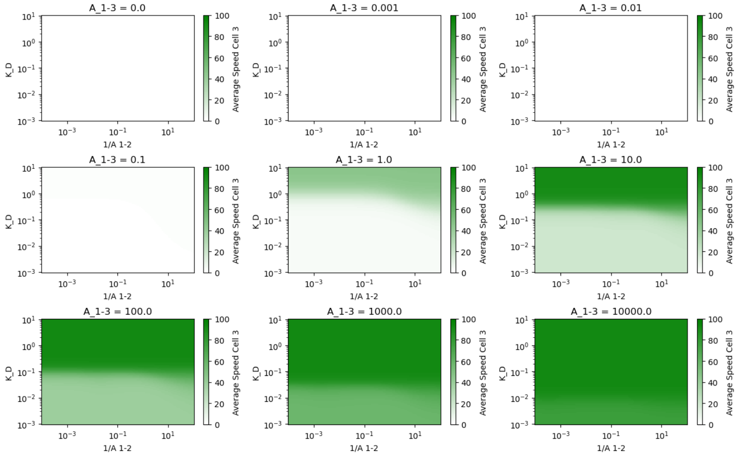

(c)

Figure 5: Notch activation speed as a function of K_D and A_{1-2} while keeping A_{1-3} fixed for a) cell 1, b) cell 2, and c) cell 3.

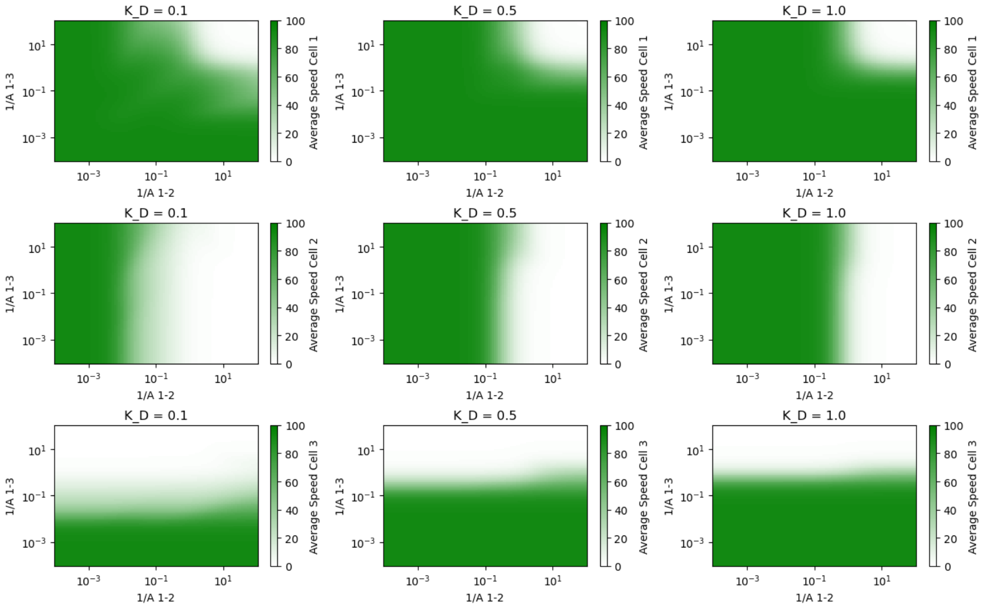

(a)

Figure 6: Notch activation speed as a function of A_{1-2} and A_{1-2} while keeping K_D fixed for cell 1, 2, and 3.

Delta-positive Notch-positive cells observed in intestinal stem cells after injury. The addition of another cell only effectively changes the K_N of the two-cell model, and this change is not sufficient to induce a high-Delta high-Notch state. However, the addition of other cells can increase the speed of Notch activation. Since increasing K_D also causes this effect, the combined effect of increasing K_D and cell clustering would further increase the Notch activation speed and help with tissue regeneration.

4.3 Extension to the full model

In the previous example, we assumed that the three cells are in a line so that we can remove one contact area parameter from the equation. To ensure that the conclusions from the previous section still apply when all the cells are contacting one another, we considered the full model. The difficulty with this model is the presence of four parameters, which means that to analyze this model, we need to keep at least two parameters fixed. From the examples in the previous section, the K_D values of 0.1, 0.5, and 1.0 are a reasonable range. Thus, we decided to look at cell 1 Notch and Delta levels as well as Notch activation speed as a function of A_{1-2} and A_{1-3} while keeping A_{2-3} fixed at the values of 0.1, 1.0, and 10 (Figure 7). Similar to before, for the range of different contact area parameters tested, there is no high-Delta high-Notch state without increasing K_D, emphasizing how the perturbation to K_D is necessary to produce Delta-positive Notch-positive cells. Additionally, similar to the previous section, the increase in contact area can result in higher Notch activation speed, generalizing the conclusions from the prior section.

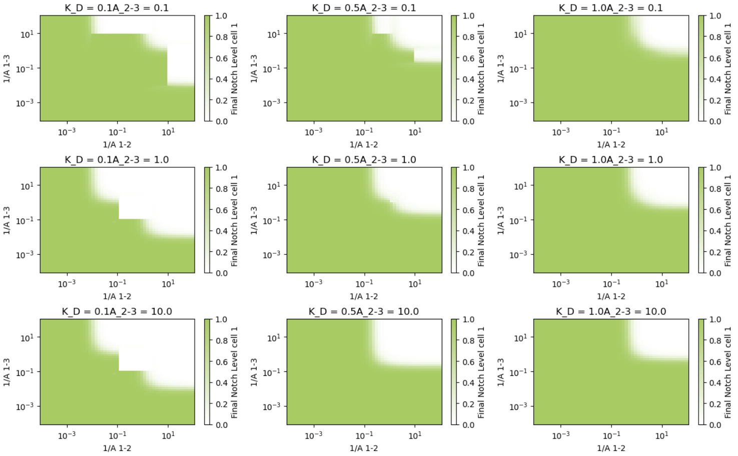

(a)

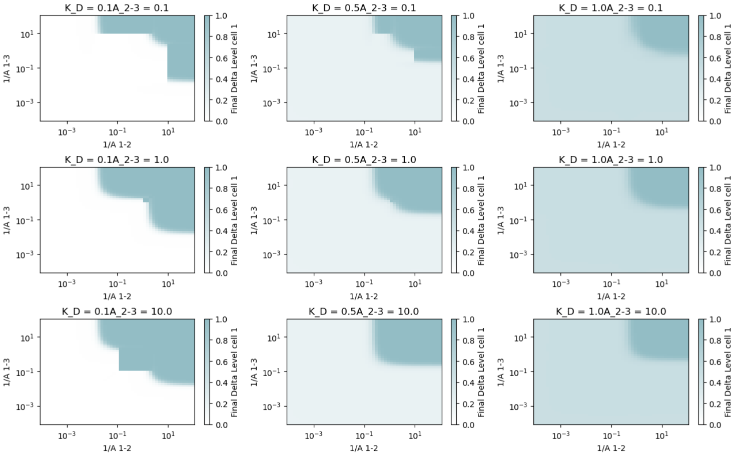

(b)

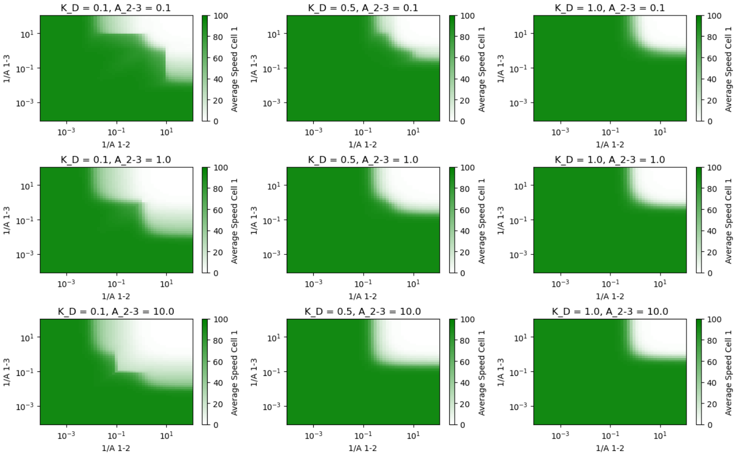

(c)

Figure 7: The equilibrium levels of a) Notch, b) Delta, and c) Notch activation speed as a function of A_{1-2} and A_{1-3} while keeping K_D and A_{2-3} fixed for cell 1.
